# Prevalence of female genital mutilation and associated factors among women and girls in Africa: a systematic review and meta-analysis

**DOI:** 10.1186/s13643-023-02428-6

**Published:** 2024-01-12

**Authors:** Asteray Assmie Ayenew, Ben W. Mol, Billie Bradford, Gedefaw Abeje

**Affiliations:** 1 Department of Obstetrics and Gynaecology, Monash University, Melbourne, Victoria Australia; 2 Department of Midwifery, Bahir University College of Medicine and Health Science, Bahir Dar, Ethiopia; 3 Department of Reproductive Health, Bahir Dar University, Amhara, Ethiopia

**Keywords:** Female genital mutilation, Women, Girls, Africa, Factors, Systematic review and meta-analysis

## Abstract

**Background:**

Female genital mutilation (FGM) has zero health benefits. It can lead to short- and long-term risks and complications, including physical, sexual, and mental health and well-being of girls and women. It is a worldwide public health issue with more than 80% prevalence in Africa. It is a global imperative to strengthen work for the elimination, and the United Nations Sustainable Development Goal (SDG) strives to eliminate FGM and monitor the progress made. However, one of a challenge in tracking progress is establishing baseline prevalence data within regions and countries. Therefore, this review aimed to pool the prevalence of FGM in Africa and identify the promoting factors among women and girls.

**Methods:**

This review was conducted according to the PRISMA checklist guideline. Both published and unpublished studies conducted from 2012 onwards were eligible. Studies written in non-English languages were excluded. To retrieve relevant studies; PubMed/Medline, Google Scholar, Science Direct, African Journals Online databases, and African Index Medicus (AIM) were searched using a combination of searching terms. The Newcastle-Ottawa Assessment Scale (NOS) tool was used to assess the quality of each included study. The Cochran’s *Q* chi-square and *I*^2^ statistical tests were used to evaluate the heterogeneity of the included studies. The Funnel plot and Egger's regression test (*p* value < 0.05) were used to evaluate meh publication bias. We used STATA for analysis and the overall and subgroup pooled effect size was estimated using the random effect model with DerSimonian and Laired pooled effect method. The overall prevalence of FGM and the adjusted odds ratio (AOR) with 95%CI (confidence interval) for contributing factors were calculated and presented using a forest plot.

**Result:**

This study included 155 primary studies conducted on the prevalence and/or factors associated with FGM in Africa. The pooled prevalence of FGM was 56.4% (95%CI 49.7–63.6). The primary factors promoting the practice of FGM were family history of circumcision (AOR = 13.71, 95%CI 9.11−20.62), being a Muslim religion follower (AOR = 3.51, 95%CI 2.61−4.71), poor wealth index (AOR = 1.38, 95%CI1.27−1.51), higher age (AOR = 2.95, 95%CI 2.49−3.38), not attending formal education (AOR = 3.28, 95%CI 2.62−4.12), and rural residency (AOR = 2.27, 95%CI 1.84−2.80).

**Conclusion:**

The prevalence of FGM in Africa was found to be high. This study also observed a variation in FGM prevalence across regions and countries and a slight temporal decline over the study period. As the global community enters the final decade dedicated to eliminating FGM, there remains much to be done to achieve the elimination goal.

**Supplementary Information:**

The online version contains supplementary material available at 10.1186/s13643-023-02428-6.

## Background

Female genital mutilation (FGM/C), also known as circumcision or genital cutting, is a gender-based violence that threatens the health and well-being of millions of infants, girls, and women across the globe and dims their future [1].

FGM is a practice carried out on infants, girls, and women involving the cutting or alteration of the external genitalia. It includes piercing, cutting, and removing the clitoris or stitching of the labia majora for non-medical reasons [2]. The age at which women and girls may experience FGM differs across communities, countries, and cultural groups. It is usually performed at the youngest age, from 7 to 8 days to 15 years old, but it could be practiced at any age [3]. FGM is a global public health concern. The United Nations (UN) estimates that over 200 million women and girls globally have experienced female genital mutilation (FGM) [4]**.** FGM is widely practiced in most African countries, certain Asian countries, and the Middle East. It is almost universal in some countries including Somalia, Djibouti, and Guinea with a prevalence of more than 90% [5, 6]. Moreover, its consequences extend beyond regions where FGM is highly prevalent but also to those residing in various parts of the world [7]. Evidence suggests that FGM exists in places including Saudi Arabia, Colombia, the United Arab Emirates, and Malaysia [8, 9]. Studies have revealed the existence of FGM in Australia, India, Israel, Indonesia, and the USA. This occurrence is attributed to migrants who bring their socio-cultural practices to these nations [1, 10–13].

The World Health Organization (WHO) identified four major classifications of FGM based on the extent of practice and genital anatomy involved (Type I, II, III, and IV) [14]. The practice and continuation of FGM are motivated by a complex of interweaved factors including socio-cultural, economic, geographical, and religious reasons [15]. In many societies, it has strong ancestral and socio-cultural roots and is considered a rite of passage to womanhood [16, 17]. Mothers and grandmothers are expected to support the practice of FGM for their daughters and granddaughters as a part of the womanhood role in the family [16]. Furthermore, FGM has been used for social cohesion and regarded by cultural custodians as a fundamental pillar of traditional practice that should be safeguarded and endorsed against the perceived threats of misguided modernizing influences [18].

Female genital mutilation has zero health benefits but leads to numerous complications for girls, women, and their families [19]. However, these common consequences may not be attributed to it and are often considered normal which can result in hiding those complications leading to a lack of seeking healthcare and underreporting [20]. The immediate complications of FGM include severe pain, hemorrhage, physical disability, shock, urinary retention, adjacent tissue trauma, surgical site infection, and the risk of viral infections from unsterilized instruments and septic environments [7, 21, 22].

The long-term complications encompass sexual, psychological, childbirth, and social problems [23, 24]. FGM also significantly impacts health-seeking behavior, i.e., access to essential services such as gynecological examinations and screenings, antenatal care, labor delivery services, and treatment for genitourinary system conditions [25, 26]. In cases of a severe form of FGM (type ӀӀӀ FGM), further surgery may be required, such as de-infibulation (opening surgery) for sexual intercourse or during childbirth. For some women, re-infibulation may also be practiced, resulting in repeated procedures with additional consequences, including physical, psychological, and sexual dysfunction [27, 28].

Moreover, FGM places a significant economic burden on global and national economies. Recent estimates indicate that the overall cost of addressing the health impacts and consequences of FGM would reach 1.4 billion dollars annually on a global scale [29].

This translates to nearly 10% of yearly health expenditure for individual countries, with some nations experiencing costs as high as 30% [30, 31]. For more than two decades, significant efforts have been made at the community, national, and international levels to eliminate the practice. There is a global initiative to end FGM but the overall reduction in prevalence rate is not sufficiently significant. The progress is uneven, varying across countries and regions [32].

The presence of factors that promote FGM and challenges in establishing accurate and baseline data within countries and regions are some of the barriers to the elimination progress. This study aimed to provide accurate and recent prevalence estimates of FGM and associated factors among African women and girls from studies conducted from 2012 onwards.

## Method

This review followed the latest Preferred Reporting Items for Systematic Reviews and Meta-Analyses (PRISMA 2020) guideline checklist [33] (Additional file 1). The study protocol has been registered in (PROSPERO) “CRD42022306730”.

### Eligibility criteria

#### Inclusion

• Setting/context: This review included all studies conducted on the prevalence of FGM and associated factors in Africa.

- Study design: all observational studies
- Language: articles conducted and reported in the English language were included
- Outcomes: the prevalence of FGM was the primary outcome, and associated factors were the second outcome variables
- Publication year: articles conducted between 2012 and August 2022 were considered for this study. The reason for using 2012 as a cut-off point stems, from 2012 a milestone resolution call was adopted by the United Nations Assembly for the international community to intensify efforts to end FGM [34]. Additionally, February 6th was dedicated as the international day of zero tolerance for FGM, aiming to amplify and direct the efforts to eliminate this practice during 2012. The African Union also committed to ending FGM in one generation [35] during this time.

### Exclusion criteria

- Studies on the consequences of FGM, qualitative studies, case reports, case series, commentaries, and editorials were excluded. Additionally, studies focused on knowledge and attitude without reporting data on the outcomes of interest, program evaluation studies, and studies available only as abstracts were excluded.

### Outcomes of measurement

The prevalence of FGM and associated factors were the first and second outcome variables, respectively. Of the included studies, the pooled prevalence and the adjusted odds ratio were calculated for common factors associated with FGM. The most common factors included in this review were family history of FGM, being a Muslim religion follower, poor wealth index, higher age, having no formal education, and rural residency.

*Female genital mutilation (FGM)* is the practice of cutting or removing a partial or total female external genitalia for non-medical reasons. FGM is usually performed between infancy and the age of 15, although it can be carried out at any age [36]. FGM is classified into four categories based on the extent of the practice and anatomy involved.

*Type Ӏ*, also called clitoridectomy or “Sunna”, is a procedure of removing a clitoral hood. The clitoris may or may not be removed partially or totally.

*Type ӀӀ*, also referred to as excision, entails a partial or total removal of the clitoris and the inner labia (labia minora) with or without excising the outer labia (labia majora).

*Types ӀӀӀ*, known as infibulation or ”pharaonic” circumcision, is a narrowing of the vaginal orifice by creating a covering seal or repositioning the labia together with or without excision of the clitoris. It has two subtypes (ӀӀӀ a and b) and is the extreme form of FGM.

*Type IV*, includes all other practices on the female external genitalia, including pricking, piercing, incision, scraping, cauterizing, or using corrosive substances designed to scar and narrow the vaginal.

*De-infibulation* is a surgical procedure of opening the sealed vaginal orifice who has undergone infibulation (Types ӀӀӀ). The procedure is often performed to facilitate sexual intercourse and childbirth or address medical conditions such as hematometra.

*Re-infibulation* is a procedure involving the re-stitching or sewing of the opened vaginal orifice after being de-infibulated, i.e., they may undergo a series of repeated infibulations and de-infibulations.

### Information sources and search strategy

Before commencing, different databases, including PROSPERO, were searched to check if there were ongoing projects or published research studies on our research topic. We have searched electronic databases: PubMed/Medline, Google Scholar, Web of Science (WOS), Science Direct, African Journals Online databases, African Index Medicus (AIM), and WHO websites. We used different search terms and strings for different databases. The search terms were formulated based on the Medical subject headings (MeSH) thesaurus using different key terms on FGM**,** including “Female genital mutilation” OR “female genital cutting” OR “circumcision” OR “infibulation” OR “Sunna” AND/OR “types” OR “clitoridectomy” OR “excision” AND/OR “prevalence” AND “girls” OR “women” OR “reproductive age women” OR “daughters” OR “female” OR “infants” AND/OR “factors”, OR “determinant” AND related “sub-Saharan Africa” OR “Africa”. AND/OR Boolean operators were used to retrieve all relevant articles meticulously**.** Moreover, grey literature was searched from research repositories and online libraries, and a secondary search technique called “footnote chasing” was used to identify additional articles from the included articles.

### Risk of bias and quality assessment

The Newcastle-Ottawa Quality Assessment Scale (NOS) was used to assess the quality of eligible studies [37]. In customizing the NOS scale, representativeness, sample size, response rate, ascertainment of the exposure, assessment of the outcome, and the statistical test were taken into account. Finally, the result of each study was categorized as poor, fair, and good quality based on the NOS (0–2, 3–5, 6–9) results, respectively.

### Statistical analysis

Articles identified through electronic database searching were exported to Endnote 20, where duplicated studies were then eliminated and exported to Microsoft Excel. The primary outcome measure focused on the prevalence of FGM, while the secondary outcome examined the factors associated with FGM. The overall and subgroup pooled effect sizes were calculated using the random effect model with DerSimonian and Laired pooled effect method [38]. To assess the heterogeneity of the included studies Cochran’s *Q* chi-square and *I*^2^ statistical tests were employed [39]. Heterogeneity was interpreted with *I*^2^ values of 0%, 25% , 50% , and 75% indicating no heterogeneity, low, moderate, and high heterogeneity, respectively [40]. Publication bias was evaluated using a funnel plot and Egger's weighted regression test [41]. All the analyses were performed using STATA software. A significant level of 0.05 was adopted.

## Results

### Study selection

**Fig. 1:**
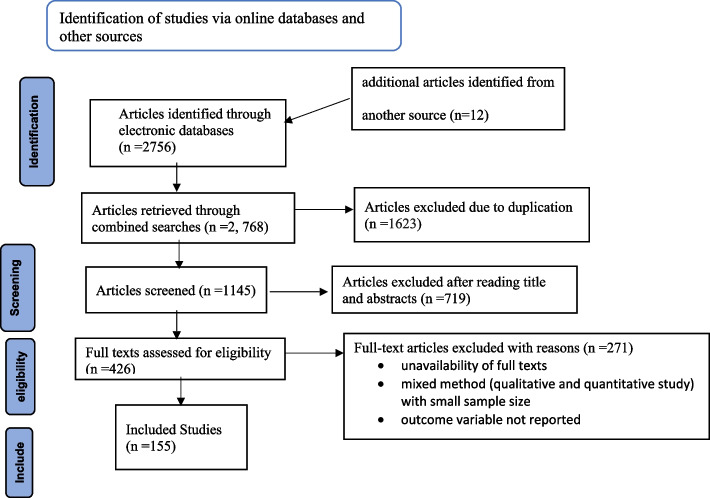
PRISMA flow diagram, 2023

We identified 2768 studies from different databases. After duplicates were expunged, 1145 studies remained, and 719 were excluded after reviewing the titles and abstracts. Of 426 full-text articles reviewed, 271 studies were excluded for different reasons, i.e., the outcome of interest not reported, inaccessibility of full texts, and mixed method (qualitative and quantitative study with small sample size. Finally, 155 primary studies conducted on the prevalence and/or factors associated with FGM were included (Fig. 1).

### Study characteristics

**Fig. 2:**
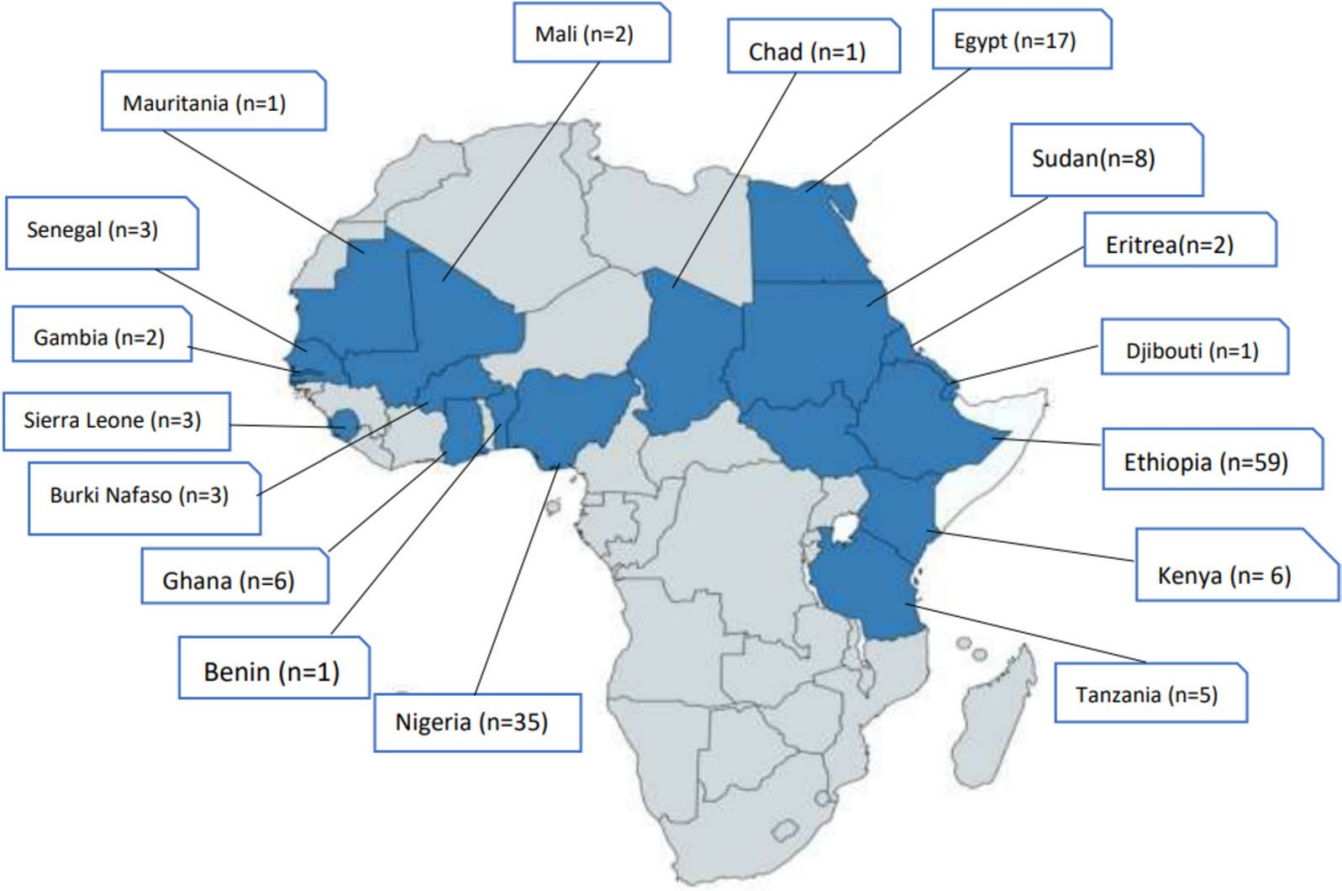
A map showing the included studies by countries

This review included different studies from different African countries, such as 59 studies from Ethiopia, 35 studies from Nigeria, and seventeen studies from Egypt (Fig. 2). Different study designs and settings were included, i.e., hospital-based, community-based, school-based, and demographic health survey studies. Furthermore, some studies reported the prevalence and associated factors of FGM on women only, some reported girls only, and others reported both women and girls (Additional file 2).

### Risk of bias assessment

The NOS tool was used to assess the quality of each eligible study. In this review, the included studies were observational (cross-sectional and case-control). Hence, NOS for cross-sectional and case-control studies were used. Adding the NOS criteria, all studies scored seven and more were considered as having good quality and included in this review (Additional file 3).

## Meta-analysis

### Publication bias and sensitivity analysis

**Fig. 3:**
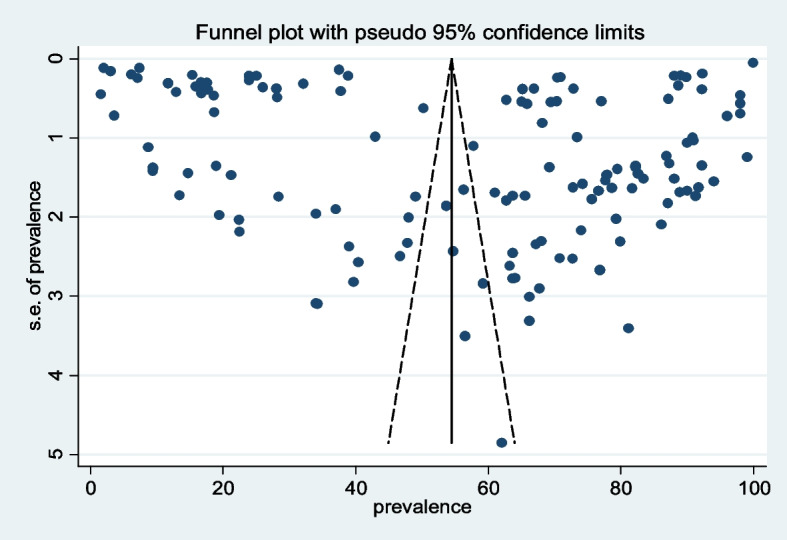
Funnel plot showing publication bias of the included studies

We used Egger’s test (*p* value = 0.150) to assess the publication bias within the included studies. Additionally, the asymmetric distribution of the prevalence of FGM was evaluated by using subjective evaluation of the funnel plot and revealed no publication bias (Fig. 3). We executed a leave-one-out sensitivity analysis and suggested that the result was not dependent on a single study. Therefore, the analysis for trim and fill was not further computed.

### The pooled prevalence of FGM in Africa

We excluded one study from the pooled prevalence estimate of FGM because it did not report the prevalence of FGM but included it in the factor analysis. Therefore, a total of 154 primary studies were analyzed to estimate the pooled prevalence of FGM, making the pooled prevalence of female genital mutilation in Africa 56.4% (95%CI 49.7−63.6).

**Fig. 4:**
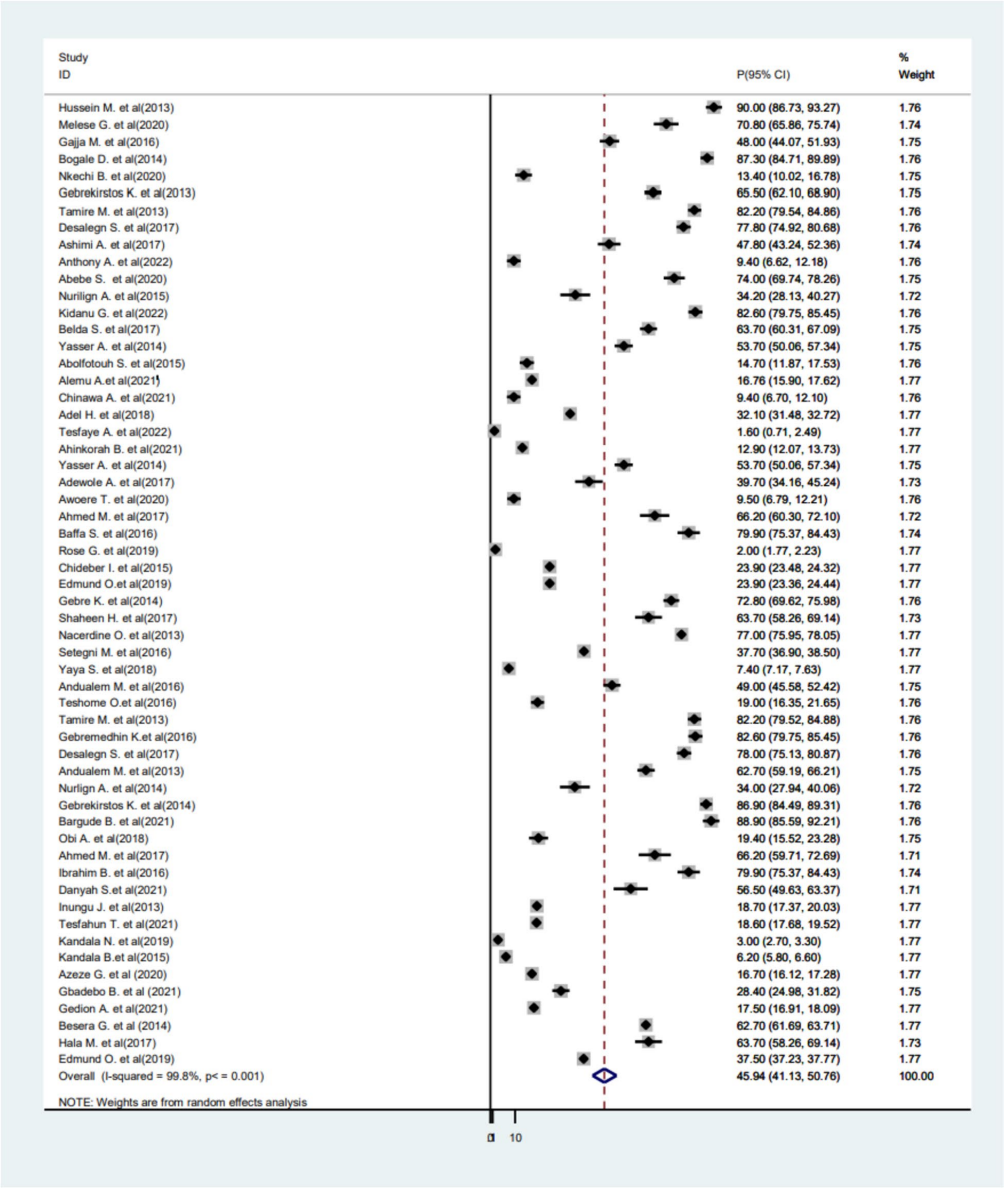
Forest plot displaying the pooled prevalence of FGM among African girls

The prevalence of FGM among girls and women was pooled separately. Among the included studies, 57 studies reported the prevalence of FGM among girls and the pooled prevalence became 45.9% (95%CI 41.13−50.76) (Fig. 4). Additionally, the pooled prevalence of FGM among women was 62.8% (95%CI 55.1−70.58).

### Types of FGM

**Fig. 5:**
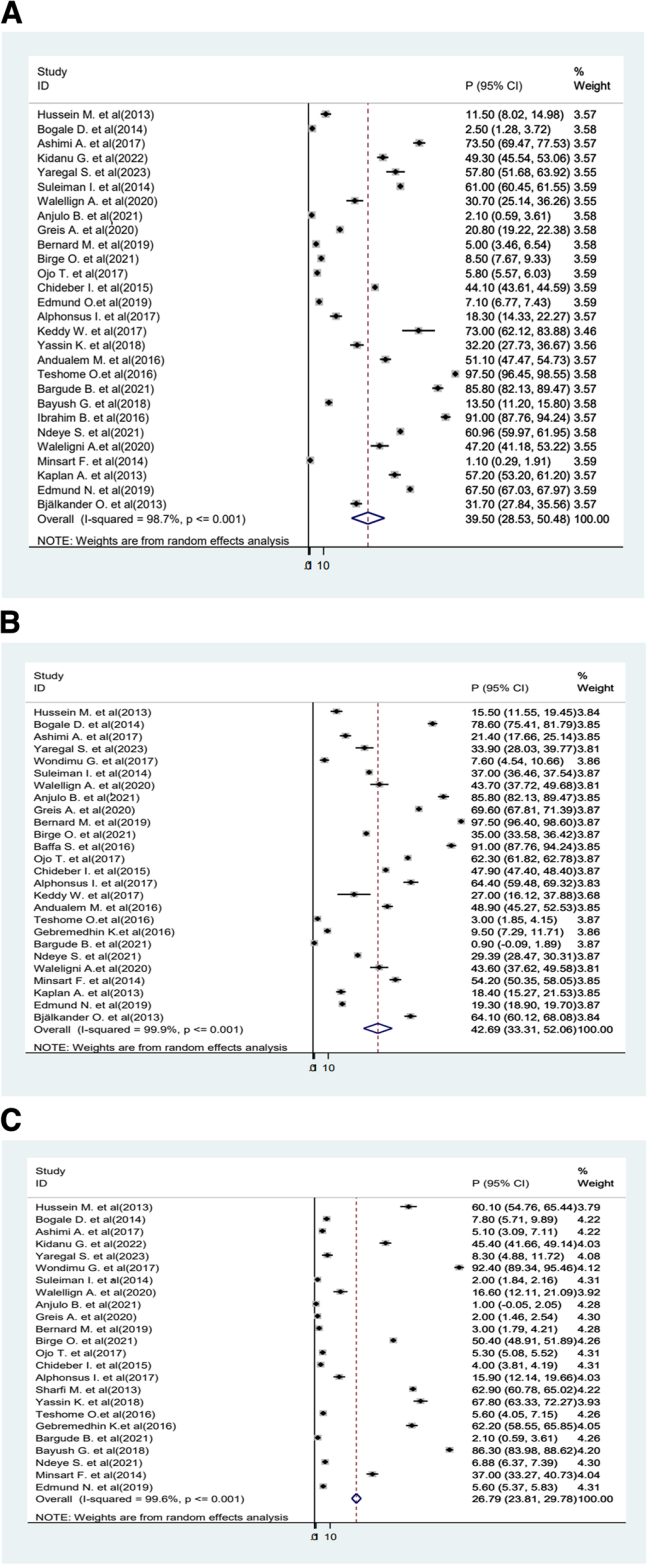
**a**–**c** Forest showing the pooled prevalence of types of FGM in Africa

Analysis was conducted for each type of FGM, and the pooled prevalence was 39.5% (95%CI 28.53−50.48) for Type I, 42.9% (95%CI 33.31−52.06) for Type II, and 26.79% (95%CI 23.81−29.78) for Type III (Fig. 5a–c).

### Subgroup analysis

In this systematic review and meta-analysis, we executed heterogeneity within the included studies and revealed the presence of high heterogeneity (*I*^2^ = 99.9%, *P* value < 0.001). Thus, subgroup analysis was based on the year of study and region (Table 1).

**Table 1:** Subgroup analysis based on the study area and year of publication

Variables	Subgroup	No. of studies	Model	Prevalence (95%CI)
Publication year	2013−2016	56	Random	58.9(49.3−68.3)
	2017−2019	50	Random	58.4(44.9−72.9)
	2020 and after	48	Random	51.5(42.7−60.3)
Study area	North Africa	25	Random	67.7(55−76.5)
	East Africa	74	Random	66(54.9−77.1)
	Western Africa	55	Random	39.7(33.3−46.4)

*Studies from the different countries were categorized into North, East, and West Africa based on the geographical location

### Factors associated with FGM

**Fig. 6:**
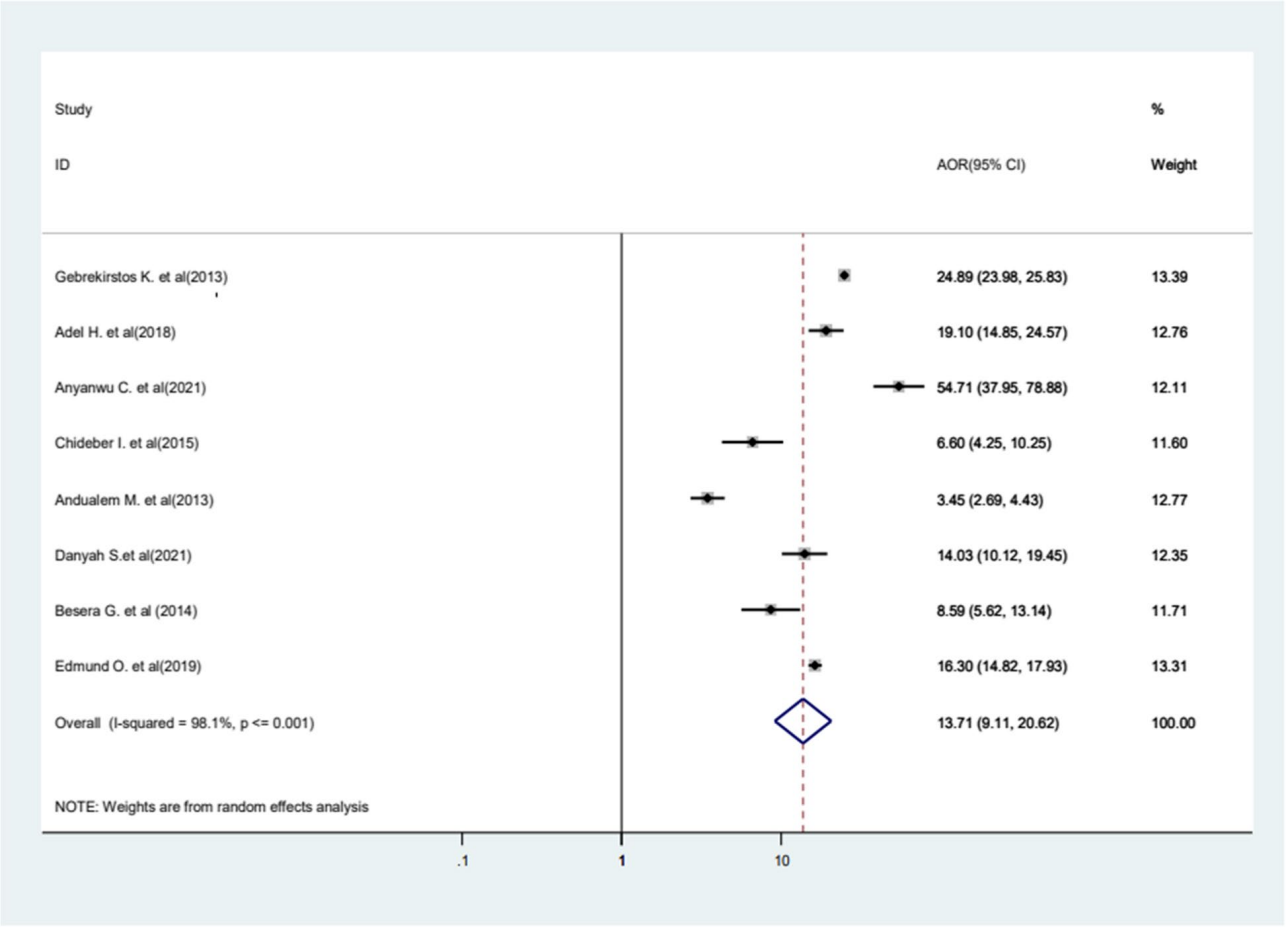
Forest plot displaying the association between being from mutilated female families (mothers and grandmothers) and FGM

This systematic review and meta-analysis examined common factors associated with FGM, such as residency, educational status, wealth index, Muslim religion, family history of FGM, and age. Notably, a significant association was found between family history of FGM and the practice in women and girls. Eight primary studies were included in this category of meta-analysis, and women and girls from mutilated female families (mothers and grandmothers) were 13.71 times more likely to undergo FGM compared to those from non-circumcised families (mothers and grandmothers) who were not circumcised (AOR = 13.71, 95%CI 9.11−20.62). The included studies were characterized as having high heterogeneity (*I*^2^ = 98.1%, *P* value < 0.001) (Fig. 6).

**Fig. 7:**
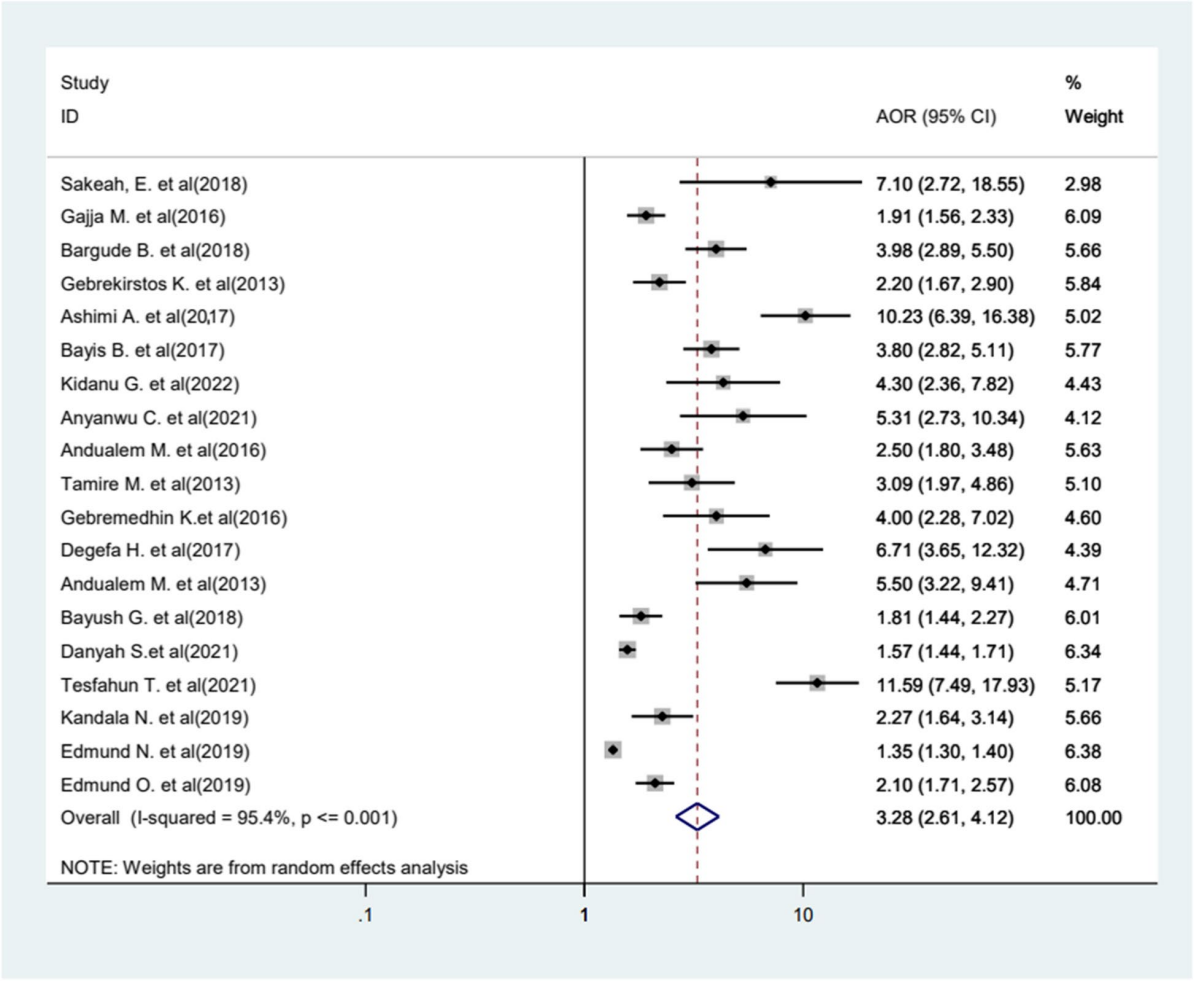
Forest plot displaying the association between educational status and FGM in Africa

**Fig. 8:**
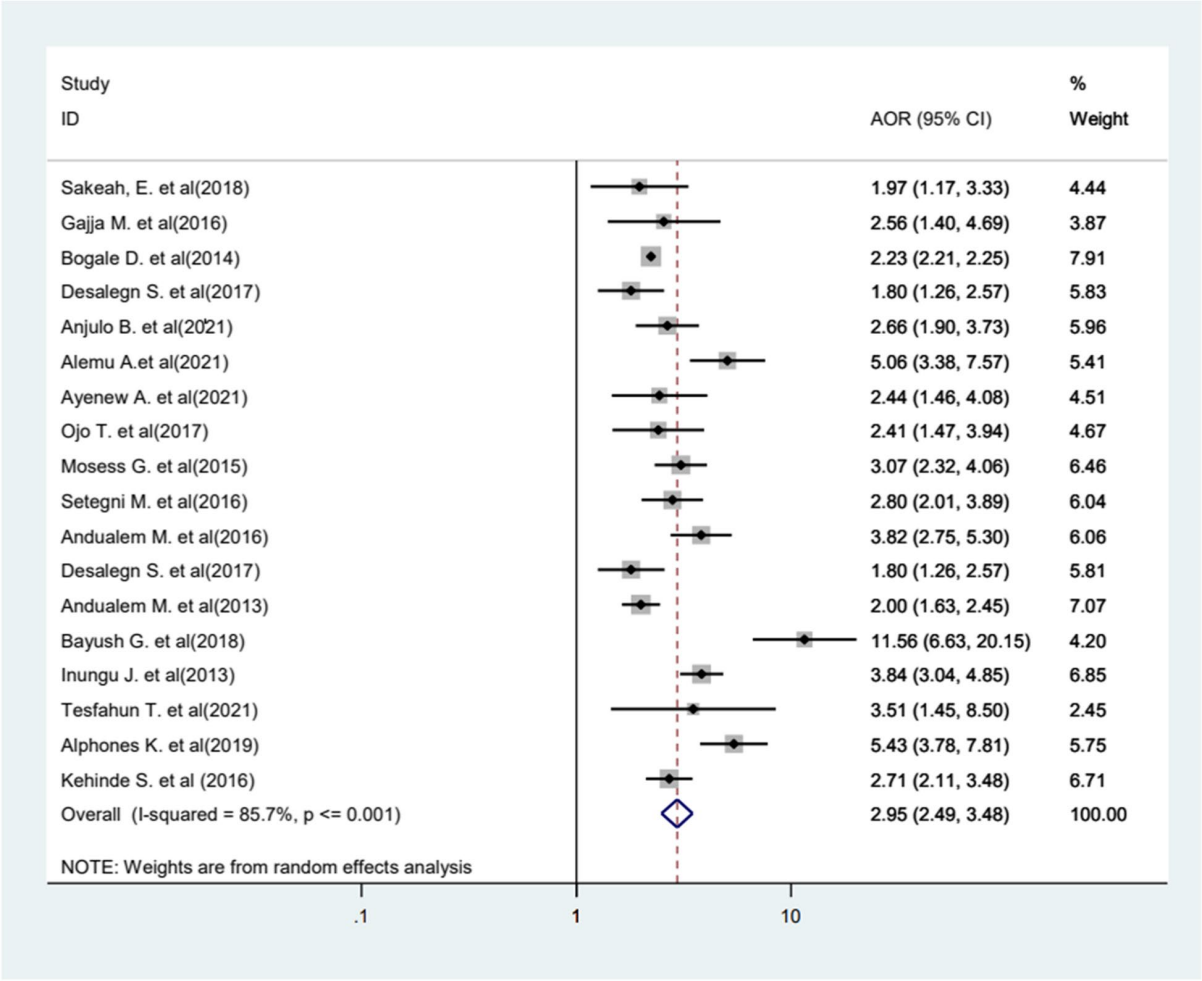
Forest plot displaying the association between age and FGM

The result of this review showed that there is a significant relationship between educational status and FGM. In this analysis, nineteen primary studies were incorporated, revealing that women and girls without any formal education had a 3.28 times higher likelihood of undergoing circumcision compared to those with formal education (AOR = 3.28, 95%CI 2.61−4.12). The included studies were characterized by the existence of high heterogeneity (*I*^2^ = 95.4%, *p* < 0.001), and we used a random effect model (Fig. 7). Furthermore, the likelihood of experiencing FGM increased by 2.95 times among older women and girls compared to younger women (AOR = 2.95, 95%CI 2.49−3.38) (Fig. 8).

**Fig. 9:**
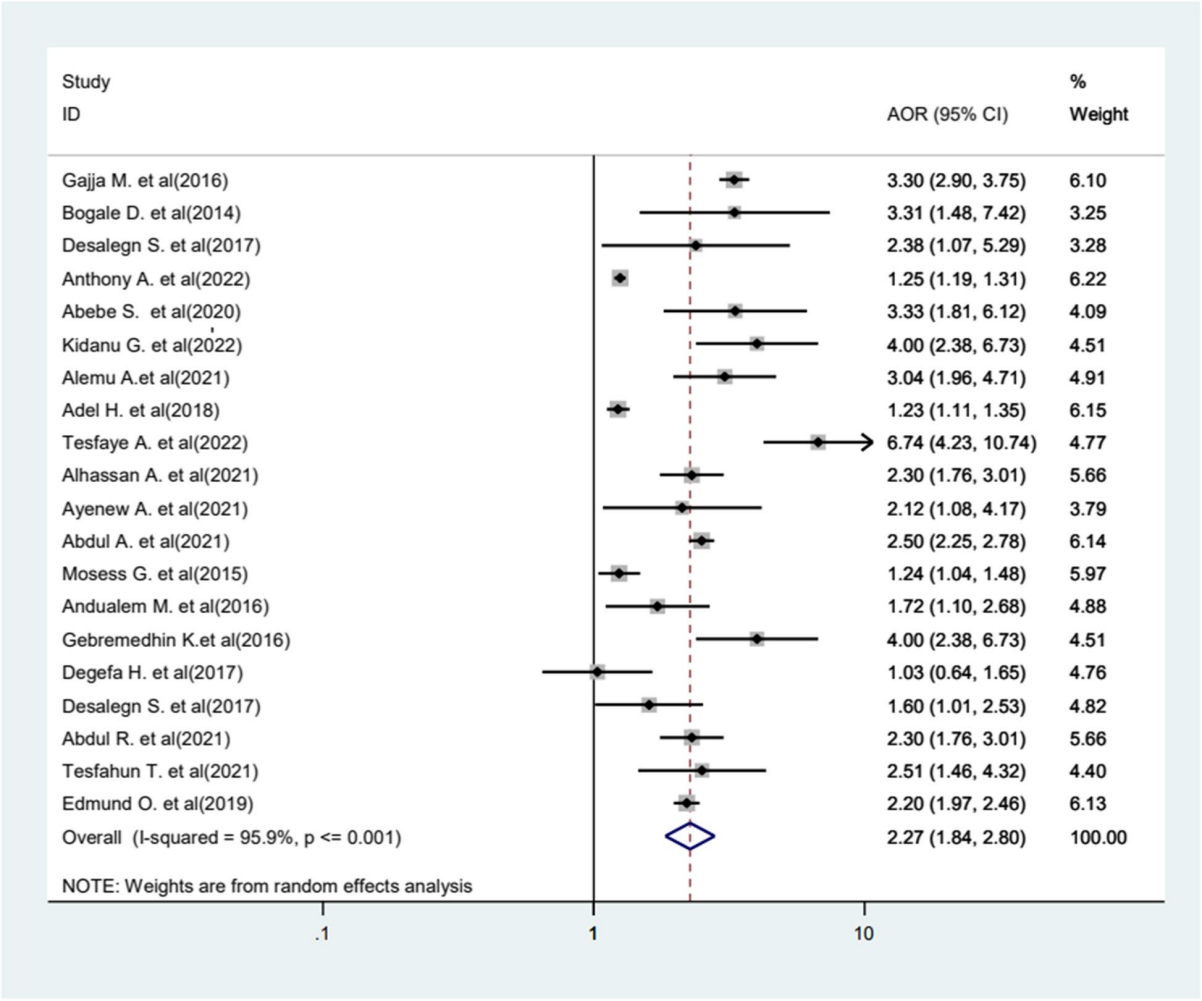
Forest plot displaying the association between residence and FGM

In this review, place of residence was significantly associated with the practice of FGM. Women and girls residing in rural areas were 2.27 times more likely to undergo mutilation compared to those living in urban areas (AOR = 2.27, 95%CI1.84−2.80) (Fig. 9).

**Fig. 10:**
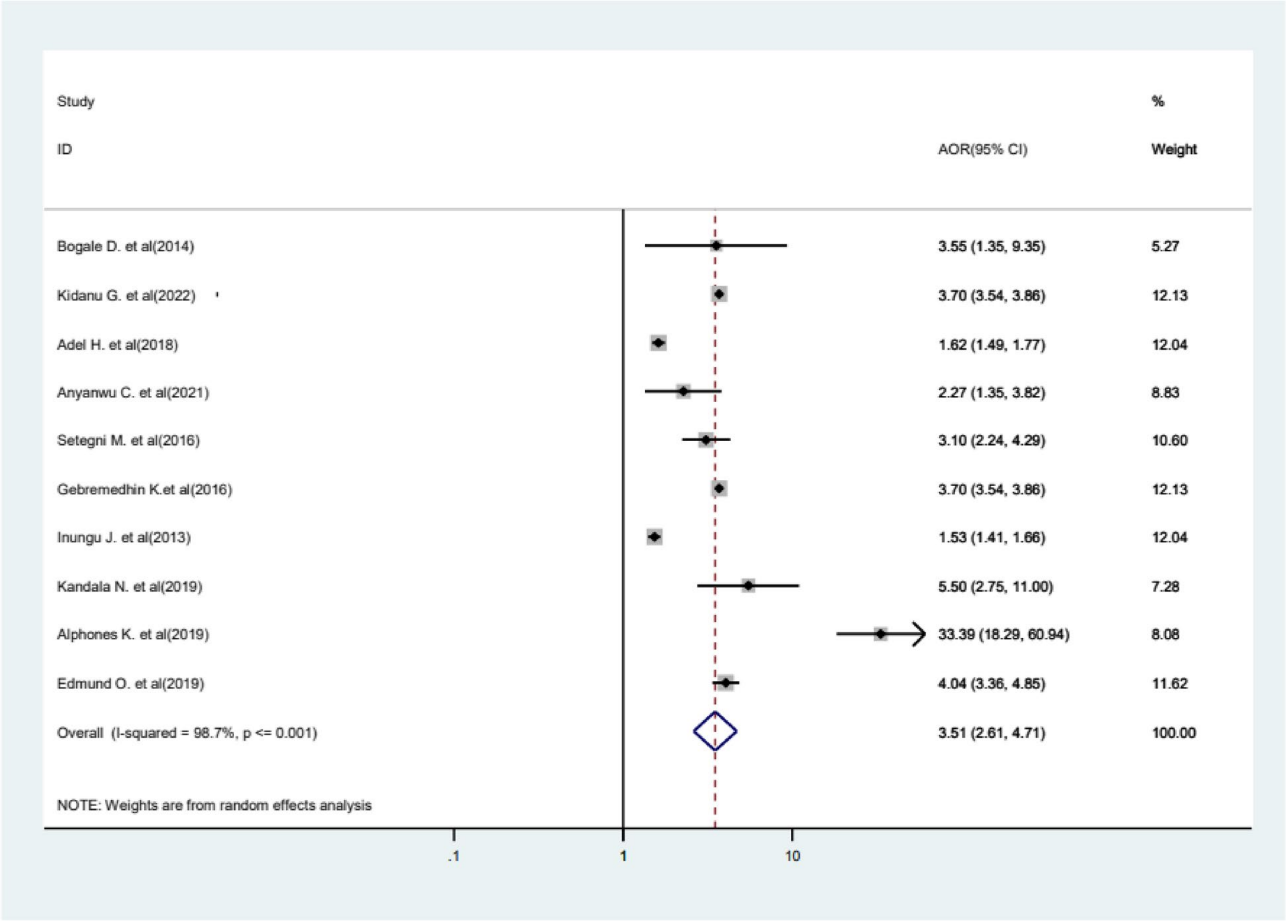
Forest plot displaying the association between being a Muslim religion follower and FGM

Following a particular religion was another determinant factor for FGM, i.e., the likelihood of FGM among Muslim followers was 3.51 times (AOR = 3.51, 95%CI 2.61−4.71) higher compared to non-Muslims (Fig. 10).

**Fig. 11:**
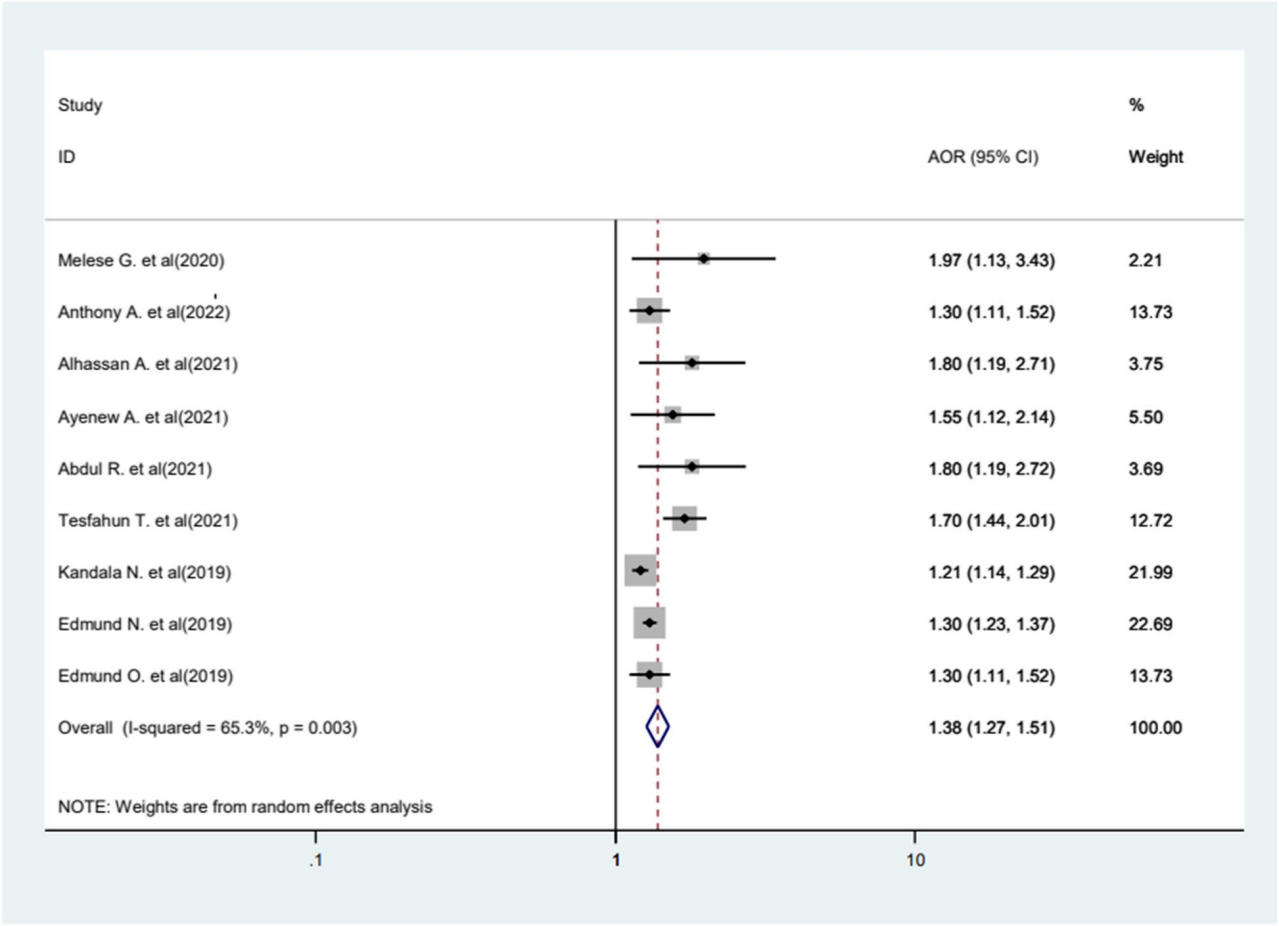
Forest plot displaying the association between wealth index and FGM

Wealth index was identified as another significant factor for FGM, with women and girls from poor index families being more likely to experience FGM (AOR = 1.38, 95%CI 1.27−1.51). The included studies were characterized by moderate heterogeneity (*I*^2^ = 65.3%, *P* = 0.03), and random effect model analysis was applied (Fig. 11).

## Discussion

This review included 155 primary studies conducted in Africa to estimate the pooled prevalence and factors associated with FGM. The pooled prevalence of FGM was 56.4% in Africa. This estimate was higher than estimates from other studies conducted in Europe 16.3% [42], a global study on FGM, 40% [43], and Sub-Saharan African countries (DHS), 51.7% [44]. The possible reason could be the difference in the study design, including countries, sample size, and study participants. The other possible reason might be differences in socio-demographic, socio-cultural variation, religion, wealth index, commuted literacy, educational status, and access to media like listening to radio/watching TV among populations in existing studies. Our results represent a comprehensive estimate of the contemporary prevalence of FGM in Africa. The persistently high prevalence of FGM among girls in Africa has supreme importance in evaluating the progress and achieving the goal of Ending FGM. This finding also has implications for clinical and institutional policymakers working on FGM and sexual and reproductive health in Africa and globally.

Although the pooled prevalence of FGM was high, we observed an overall decrement of FGM of 7.5% in later research conducted in 2020 and after (51.5%) compared to earlier surveys conducted from 2013 to 2016 (60.1%). This result is in line with a study conducted on secular trends of FGM in Africa and European countries [12], the WHO report [45], and a global survey [43]. The decline in FGM prevalence could be attributed to international pressure and advocacy against FGM, the presence and effectiveness of local organizations, and community-led initiatives in raising awareness about FGM [46]. The other possible reason might be that many African countries are obliged to comply with international conventions for the rights of women and girls, banning FGM and applying legal measures to prevent the practice [47]. However, despite the decrement in the prevalence of FGM over time, in some countries, it remains nearly universal or as common today as it was many years ago, indicating that progress needs to be accelerated to meet the SDG target 5.3 by 2030 [48].

The prevalence of FGM varies from country to country and between regions in Africa. The pooled prevalence of FGM was 67.7%, 66%, and 39.7% in the Northern, Eastern and Western Africa respectively. These findings are consistent with a previous study by UNICEF [49] and other studies conducted on FGM differences between countries and regions [12, 50, 51]. The possible explanation could be the prevalence of FGM is influenced by multiple factors such as socio-economic, cultural, religious, commitment to lower or eliminate the practice, and rapid population growth [12]. Additionally, demographic factors such as ethnicity, geographic factors like proximity to borders, and cultural influences from neighboring countries may also shape the prevalence rate.

The prevalence of FGM was found to be higher among women (62.8%) compared to girls (45.9%). This result is in line with findings from other studies on FGM among women and girls [3, 12, 44, 52]. The reason behind this could be due to changing attitudes within communities and is a piece of evidence of the success of national and international investment to reduce or end FGM, i.e., FGM is less common in younger generations [53, 54].

Among the types, Type II was the most common type, with a pooled prevalence of 42.9%, and the lowest was Type III, 26.79%. This result is supported by studies [3, 50, 53, 55] conducted on types of FGM. Understanding the distribution of FGM types can help tailor interventions and healthcare services to address the specific needs and health consequences associated with each type.

This study revealed that women and girls from families with a history of FGM were 13.71 times more likely to undergo FGM compared to their counterparts. This finding aligns with previous research, including the WHO report [56], a systematic review [57], and studies conducted in the African context [58–60], as well as the work of Pashaei T. et al. [61]. The possible reason may be females from a family member who has undergone FGM can be strongly influenced by their elders about the cultural traditions including FGM which in turn promote the practice.

Women and girls residing in rural areas were found to be 2.27 times more likely to undergo mutilation compared to those in urban areas. The findings are consistent with studies conducted in SSA [44, 62], a systematic review in Europe [57, 63], Africa [64], and Eastern Africa [65]. The possible reason for this may be in rural communities where educational opportunities are often limited and access to information about FGM and its consequences is scarce. Additionally, in rural areas, FGM has been used to maintain socio-economic ties and enhance marriageability, further perpetuating the practice [12, 57, 66, 67].

Women and girls without formal education have a 3.28 times higher likelihood of undergoing FGM compared to those who had formal education. The result is in line with studies conducted in SSA [44], a study of FGM and education [66], a systematic review [57], United Nations Children’s Fund data [68], and another study in Africa [64]. The possible reason might be women and girls with no formal education have limited access to information and counseling about FGM and its consequences and are, therefore, more likely to adhere to traditional practices [44, 69]. Additionally, a lack of education can lead to limited decision-making power, making it harder for them to challenge or reject the practice.

This study also revealed that women and girls from poor wealth index have a 1.38 times higher risk of undergoing FGM compared to those from wealthier households. The result is in line with other studies [29, 44, 60, 70, 71] that have explored the association between wealth status and FGM. The possible reason might be wealth is often intertwined with other social determinants, such as a place of residence and education, which are associated with FGM [44, 72]. Additionally, women and girls are more prone to harmful traditional practices, including FGM, because of their economic dependency and poverty [73].

Followers of the Muslim religion were 3.51 times more likely to undergo FGM compared to those from other religious backgrounds. The result is in line with other studies conducted in SSA [44], Africa [74–79], and a systematic review [57] that have explored the association between FGM and religion. FGM is not mentioned as a religious requirement, and the observed association between FGM and the Muslim religion can be attributed to the cultural entanglement of the practice with certain religious practices [74].

Furthermore, older women and girls had greater odds of experiencing FGM by 3.19 times compared to younger ones. The result was supported by other African studies [58, 80, 81]. This might be because older females may be more deeply rooted in traditional practices due to a lack of education and long-standing adherence to cultural norms that shape group identity and heritage [82]. This outcome suggests the prevalence of FGM is decreasing in younger generations, possibly as a result of interventions, better awareness about FGM, and change of attitude within the community [83].

## Strength and limitation

One of the strengths of this study is providing the prevalence estimates likely to reflect the recent prevalence of FGM and associated factors in Africa by including studies from 2012 onwards.

Additionally, the data was extracted using a pre-determined tool, and quality assessment was conducted using the NOS assessment tool, leading to the inclusion of studies with moderate and high quality to enhance the findings’ reliability. However, there are some limitations to consider; only papers conducted in the English language were included, and not all countries in Africa were included because of the unavailability of recent studies. Some studies were excluded because of the unavailability of full text, mixed method (qualitative and quantitative study) with small sample size to determine the prevalence, and the outcome variables were not reported. There was high heterogeneity among the included studies, and considering this limitation, a random-effect model was used to compute the pooled prevalence of FGM.

## Conclusion

The pooled prevalence of FGM is high in Africa. FGM continues to persist due to several factors, including a family history of circumcision, being a Muslim religion follower, poor wealth index, higher age, lack of formal education, and rural residency. We are in the final decade to make zero new cases of FGM, and comprehensive interventional efforts are recommended to achieve the elimination goal. Awareness creation, providing educational opportunities, especially for rural residents, empowering women, and addressing their economic independence would be crucial to combat FGM. Furthermore, breaking the cycle of intergenerational transmission by engaging with families, men, and elders to foster dialogue and challenge harmful beliefs about FGM can be helpful in lowering or eliminating it in Africa. Engaging with religious leaders is essential to promote understanding and support for ending FGM while respecting cultural and religious sensitivities.

### Supplementary Information

**Additional file 1.**  PRISMA 2020 Checklist.

**Additional file 2:** **Table 1.** Characteristics of included studies.

**Additional file 3:** **Table 1.** Risk of bias assessment for cross-sectional studies – Newcastle-Ottawa Scale (adaptation). **Table 2.** Risk of bias assessment for cohort studies – Newcastle-Ottawa Scale (adaptation).

## Data Availability

The majority of data related to this review is available within this paper, and further information may be obtained from the corresponding author upon a reasonable request.

## Abbreviations

FGM: Female Genital Mutilation
NOS: The Newcastle and Ottawa Quality Assessment Scale
PRISMA: Preferred Reporting Items Systematic Review Meta-Analysis
SDG: Sustainable Development Goals
SSA: Sub-Saharan Africa
