# Supplementary material for: Prevalence of female genital mutilation and associated factors among women and girls in Africa: a systematic review and meta-analysis
Source: Syst Rev. 2024 Jan 12;13:26. doi: 10.1186/s13643-023-02428-6 (PMC10785359; doi:10.1186/s13643-023-02428-6)
Supplement: Supplementary file 2 — Additional file 2: Table 1. Characteristics of included studies. [file 13643_2023_2428_MOESM2_ESM.docx]

Additional file 1

Table 1: characteristics of included studies

| Authors (year of study) | Study area | Study design | Study participants |
| --- | --- | --- | --- |
| Sakeah, E. et al(2018) (1) | Ghana | Community based cross sectional | Reproductive age women |
| Hussein M. et al (2013)(2) | Ethiopia | Community based cross-sectional | Women 15-49 years |
| Gajja M. et al (2016) (3) | Ethiopia | community based Cross sectional | daughter under 5 years of age |
| Gajja M. et al (2016) (3) | Ethiopia | community based Cross sectional | mothers who had at least one daughter |
| Bargude B. et al (2018)(4) | Ethiopia | community based Cross sectional | Reproductive age women |
| Bogale, D. et al (2014) (5) | Ethiopia | community based cross sectional | Reproductive age women |
| Bogale, D. et al (2014)(5) | Ethiopia | community based cross sectional | Last daughter less than 15 years old |
| Nkechi B. et al (2020)(6) | Nigeria | community based cross sectional | Child bearing age women |
| Setegni M. et al(2016)(7) | Ethiopia | Demographic Health Survey | Reproductive age women |
| Setegni M. et al(2016)(7) | Ethiopia | Demographic Health Survey | daughters younger than 15 years |
| Elbendary R. et al (2021)(8) | Egypt | Hospital based cross-sectional | women in their childbearing years |
| Tamire M. et al (2013)(9) | Ethiopia | School based cross sectional | high school girls |
| Gebrekirstos K. et al (2013)(10) | Ethiopia | Community based study | children less than 5 years |
| Desalegn S. et al(2017)(11) | Ethiopia | School based cross sectional | School girls |
| Ashimi A. et al (2017)(12) | Nigeria | Hospital based cross sectional | Infants |
| Bayis B. et al (2017)(13) | Ethiopia | community based cross sectional | Reproductive age women |
| Anthony A. et al (2022)(14) | Nigeria | School based cross sectional | female students |
| Chizoma M. et al (2017)(15) | Nigeria | Hospital based Cross section | women at the post-natal clinic |
| Degefa H. et al (2017)(16) | Ethiopia | Hospital-based cross-sectional | women attending delivery service |
| Muktar S. et al (2021)(17) | Ethiopia | Community based cross sectional | Reproductive age women |
| Abebe S. et al (2020)(18) | Ethiopia | Community based mixed method | Mothers (15 to 49 years old) |
| Abebe S. et al (2020)(18) | Ethiopia | Community based mixed method | Child under the age of 15 years old |
| Nurilign A. et al(2015)(19) | Ethiopia | Community based cross-sectional | Reproductive age women |
| Nurilign A. et al(2015)(19) | Ethiopia | Community based cross-sectional | under five children |
| Kidanu G. et al (2022)(20) | Ethiopia | school-based cross-sectional | Girls 10 to 24 years old |
| Cevdet A. et al (2017) (21) | Egypt | Community based cross-sectional | Reproductive age women |
| Amari O. et al (2021) (22) | Nigeria | Community based cross-sectional | Reproductive age women |
| Wondimu G. et al (2017)(23) | Ethiopia | Institutional based cross-sectional | All nulliparous women admitted for labour |
| Belda S. et al (2017)(24) | Ethiopia | Community based cross-sectional | Daughters of reproductive age women |
| Israel J. et al (2014)(25) | Nigeria | Hospital based cross-sectional | Women visited hospital |
| Suleiman I. et al (2014)(26) | Tanzania | Hospital based cross sectional | singleton women aged 15–49 years |
| Yasser A. et al (2014)(27) | Egypt | School based cross sectional | school girls |
| Adewole A. et al (2017)(28) | Nigeria | Hospital based cross-sectional | Women |
| Adewole A. et al (2017)(28) | Nigeria | Hospital based cross-sectional | girls |
| Marwa S. et al (2020)(29) | Egypt | facility based cross-sectional | reproductive age women |
| Walellign A. et al (2020)(30) | Ethiopia | Community based cross-sectional | women age group between 15-49 |
| [Anjulo B](https://www.researchgate.net/scientific-contributions/Anjulo-Bargude-Balta-2206363533?_sg%5B0%5D=oKhNGiqN9Y60yevIYGYMluwehmEl7vLWLwHqdjhomkGBtYmtZDVVMfKIhrX-cZ2KKzkoFw8._6Cs6Zbk3AHmFDdZdUcN1miJ7jBj0xkX1IEXSoo28THIxRTchvN0N0cbywDnRCvqv4uP8LkEUDxL7jY9pu9oKg&_sg%5B1%5D=aocOZHl-_26bszLYuD1Jm1K5pUi0b2pwUyJ8UudooZNtHalQAwAz8uZhbob0Ji7V6FkXUM4.1vW_ckdEGnJr4UwXjKLl42dM9AbPaC6tfUpd9hWY8WQ0gfVtKV6NkgfhJuyV7I2QHLMujIgpfhN506-LJIjrAw). et al (2021)(31) | Ethiopia | Community based cross-sectional | Reproductive age women |
| Obi A. et al (2018)(32) | Nigeria | Facility based cross sectional | reproductive age women |
| Obi A. et al (2018)(32) | Nigeria | Facility based cross sectional | Daughters |
| Keddy W. et al (2017)(33) | Kenya | Hospital based cross-sectional | Laboring mothers |
| Greis A. et al (2020)(34) | Burki Nafaso | Community based cross-sectional | both women and adolescents aged 12–20 |
| Abolfotouh S. et al (2015 )(35) | Egypt | Hospital based cross-sectional | Medical students |
| Alemu A.et al (2021)(36) | Ethiopia | Demographic health survey | reproductive age women |
| Alemu A.et al (2021)(36) | Ethiopia | Demographic health survey | Daughters |
| Chinawa A. et al (2021)(36) | Nigeria | School based cross-sectional | Adolescent girls |
| EDHS (2016)(37) | Ethiopia | Demographic health survey | reproductive age women |
| Adel H. et al (2018)(38) | Sudan | Multiple Indicator Cluster Survey (MICS) | reproductive age women |
| Adel H. et al (2018)(38) | Sudan | Multiple Indicator Cluster Survey (MICS) | daughters |
| Tesfaye A. et al (2022)(39) | Ethiopia | Community based cross sectional | Daughters aged less than 15 |
| Tesfaye A. et al (2022) (39) | Ethiopia | Community based cross sectional | Reproductive age women |
| Ahinkorah B. et al (2021)(40) | Chad | Demographic health survey | reproductive age women |
| Tesema G. et al (2020)(41) | Ethiopia | Demographic health survey | reproductive-age women |
| Alhassan A. et al (2021)(42) | Ghana | Multiple Indicator Cluster Survey (MICS) | Women aged 15-49 years |
| Melese G. et al (2018)(43) | Ethiopia | Community based cross sectional | Daughters less than 5 years old |
| [Bernard M](https://www.researchgate.net/scientific-contributions/Bernard-Mbogo-2156379663?_sg%5B0%5D=rJsd2SbucMQHtWiESPhwjcwiAdluETZnF1kG-1bNddFlHQtEyfaWiYu-A-klFzX9QnSUsp4.RkaMqHrCoOQhAlfrL0tm1RUI0pxT5mlJpIEFpSmtU8xv2nEbHq9UclCuXkAD6jrV39xfEmQCQNJXobEiKBBFWg&_sg%5B1%5D=7usygW96NpWMOtfpO7tHa2iytbCik-ICPsRRABknVJNWEHGhTpi87-oFR_j_JGvYoB1U6r0.YD__KwApvtiIdgCLzM6_jFeU3star63FSA5vat7EG0wcB-oc_-wzyvczPbUOHVYQNPWa4igBekWxGanIUvqPCw). et al (2019) (44) | Kenya | Community based cross sectional | women of reproductive age |
| Mustafa K. et al (2020)(45) | Sudan | community based survey | reproductive age (15-49) years |
| Birge Ö. et al (2021)(46) | Sudan | Hospital based cross sectional | reproductive age (15-49) years |
| Yasser A. et al (2014)(47) | Egypt | A cross-sectional school-based | secondary school girls |
| Awoere T. et al (2020)(48) | Nigeria | School based cross sectional | female adolescents aged 13–21 years |
| Ahmed M. et al (2017)(49) | Egypt | Hospital based cross sectional | students aged 14–19 years |
| Ayenew A. et al (2021)(50) | Ethiopia | Demographic health survey | women of reproductive age |
| Hala M. et al(2017)(51) | Egypt | School based cross sectional | men |
| Baffa S. et al (2016)(52) | Nigeria | A Cross-Sectional Descriptive | women who have daughter |
| Baffa S. et al (2016)(52) | Nigeria | A Cross-Sectional Descriptive | Daughters |
| Rose G. et al (2019)(53) | Kenya | Demographic health survey | women who have daughter |
| Rose G. et al (2019)(53) | Kenya | Demographic health survey | Daughters |
| Anyanwu C. et al (2021)(54) | Nigeria | Community based cross-sectional | women of reproductive age |
| [Ojo](https://pubmed.ncbi.nlm.nih.gov/?term=Ojo+TO&cauthor_id=28641026) T. et al (2017)(55) | Nigeria | Demographic health survey | Reproductive age women |
| Chideber I. et al(2015)(56) | Nigeria | Demographic health survey | Child girl |
| Achia T. et al (2014) | Kenya | Demographic health survey | women of reproductive age |
| Melsew S. et al (2021)(57) | Ethiopia | Hospital based cross-sectional | Reproductive age women |
| Masho W. et al (2014)(58) | Egypt | Survey based study | Reproductive age women |
| Abolfotouh S. et al (2104)(59) | Egypt | Community based cross sectional | Medical student |
| Alphonsus I. et al (2017)(60) | Nigeria | hospital based cross sectional | Pregnant women |
| Alkhalaileh D. et al (2018)(61) | Egypt | Demographic health survey | reproductive-age |
| Gebre K. et al (2014)(62) | Ethiopia | Community based cross sectional | Children |
| Ahmed Y. et al (2021)(63) | Ethiopia | Community based cross-sectional | Women and girls |
| [Sharfi](https://www.sciencedirect.com/science/article/pii/S111057041300060X#!) [M](https://www.sciencedirect.com/science/article/pii/S111057041300060X#!). et al (2013)(64) | Sudan | Hospital based retrospective | Women |
| Abdul A. et al (2021)(65) | Ghana | Multiple Indicator Cluster Survey (MICS) | women |
| Besera G. et al (2014)(66) | Eritrea | Demographic and Health Survey | Reproductive age women |
| Besera G. et al (2014)(66) | Eritrea | Demographic and Health Survey | Daughters |
| Mosess G. et al (2015)(67) | Tanzania | Community based cross sectional | Reproductive age women |
| Abdurehman K. et al (2017)(68) | Ethiopia | Community based cross sectional | Reproductive age women |
| Yirgalem Y. et al (2021)(69) | Ethiopia | hospital based cross sectional study | Postnatal mothers |
| Nacerdine O. et al (2013)(70) | Mauritania | Demographic health survey | Child girls |
| Ngianga B. et al (2015)(71) | Senegal | Demographic health survey | Women |
| Ngianga B. et al (2015)(71) | Senegal | Demographic health survey | Daughters |
| Yaya S. et al (2018)(72) | Nigeria | Demographic health survey | Women |
| Kehinde S. et al (2016)(73) | Nigeria | An institutional survey | women attending the gynaecological out-patient clinic |
| Yaya S. et al (2018)(72) | Nigeria | Demographic health survey | daughters |
| Andualem M. et al (2016)(74) | Ethiopia | Community based cross sectional | Women 15-49 |
| Andualem M. et al (2016)(74) | Ethiopia | Community based cross sectional | daughters< 5 years of age |
| [Eman S](https://pubmed.ncbi.nlm.nih.gov/?term=Mohammed+ES&cauthor_id=30333019). et al (2018)(75) | Egypt | Community based cross sectional | Women and men above 18 years |
| Chinawa A. et al (2021)(76) | Nigeria | School based Cross sectional | Adolescents Attending Secondary Schools |
| Gbadebo B. et al (2021)(77) | Nigeria | Demographic health survey | Reproductive age women |
| Gbadebo B. et al (2021)(77) | Nigeria | Demographic health survey | Daughter of reproductive age women |
| Teshome O.et al (2016)(78) | Ethiopia | Demographic health survey | Reproductive age women |
| Teshome O.et al (2016)(78) | Ethiopia | Demographic health survey | Daughter younger than 12 years |
| Tamire M. et al (2013)(79) | Ethiopia | school based Cross sectional | High school girls |
| Gebremedhin K.et al (2016)(80) | Ethiopia | school-based cross-sectional | young adult (10–24 years of age) |
| Desalegn S. et al (2017)(81) | Ethiopia | Institutional based cross sectional | high school and preparatory students. |
| Andualem M. et al (2013)(82) | Ethiopia | community based Cross sectional | Daughters |
| Andualem M. et al (2013)(82) | Ethiopia | community based Cross sectional | Reproductive age women |
| Nurlign A. et al (2014)(83) | Ethiopia | community based Cross sectional | Under 5 daughters |
| Gebrekirstos, K. et al (2014)(84) | Ethiopia | Community based cross sectional | Less than 5 years children of female |
| Bargude B. et al (2021)(85) | Ethiopia | Community based cross sectional | Reproductive age women |
| Abdul R. et al(2021)(86) | Ghana | Multiple Indicator Cluster Survey (MICS) | Reproductive age women |
| Baturu M. et al (2019)(87) | Gambia | Demographic health survey | women |
| Yaregal E.et al (2014)(88) | Ethiopia | Community based cross sectional | women |
| Bernand M. et al (2019)(89) | Nigeria | Community based cross sectional | women of reproductive age |
| Nkechi O. et al (2020)(90) | Nigeria | Community based cross sectional | Child bearing age women |
| Bayush G. et al (2018)(91) | Ethiopia | Community based cross sectional | Reproductive age women |
| [Fosu](https://www.semanticscholar.org/author/M.-Fosu/52072294) M. et al (2014)(92) | Ghana | Multiple Indicator Cluster Survey (MICS) | Women |
| [Ahmed](https://www.semanticscholar.org/author/M.-R.-Ahmed/47285582), [M](https://www.semanticscholar.org/author/M.-M.-Shaaban/153353236). et al (2017)(93) | Egypt | Hospital based cross sectional | 14–19 years attending an outpatient clinic |
| [Ibrahim](https://www.semanticscholar.org/author/B.-Ibrahim/16787231) B. et al (2016)(94) | Nigeria | Community based cross sectional | Reproductive age women |
| [Shaheen](https://scholar.google.com/citations?user=TwmfE_sAAAAJ&hl=en&oi=sra) H. et al (2017)(95) | Nigeria | School based cross sectional | School girls |
| Getayeneh A. et al (2020)(96) | Ethiopia | Demographic health survey | Reproductive age women |
| Yassin K. et al (2018)(97) | Sudan | Cohort | Postpartum women |
| Ahmed M. et al (2022)(98) | Ethiopia | Demographic health survey | Reproductive age women |
| [Ibrahim](https://www.semanticscholar.org/author/B.-Ibrahim/16787231) B. et al (2016)(94) | Nigeria | Community based cross sectional | Daughters |
| Awoleke J. et al (2019)(99) | Nigeria | Community based cross sectional | Reproductive age women |
| Ndeye S. et al (2021)(100) | Senegal | Demographic health survey | Reproductive age women |
| Danyah S.et al (2021)(101) | Sudan | Community based cross-sectional | Daughters of reproductive age women |
| Azeze G. et al (2020)(102) | Ethiopia | Demographic health survey | Daughters of reproductive age women |
| [Inungu](https://www.semanticscholar.org/author/J.-Inungu/4039784) J. et al (2013)(103) | Burkina Faso | Community based cross sectional | Daughters |
| [Inungu](https://www.semanticscholar.org/author/J.-Inungu/4039784) J. et al (2013)(103) | Burkina Faso | Community based cross sectional | women |
| [Tesfahun T](https://www.semanticscholar.org/author/Tesfahun-Taddege-Geremew/150319285). et al(2021)(104) | Ethiopia | Demographic health survey | Girls aged 0 to 14 |
| [Akpan](https://www.semanticscholar.org/author/B.-Akpan/122181332) B. et al(2019)(105) | Nigeria | Hospital based cross sectional | Antenatal care attendees |
| Waleligni A.et al(2020)(106) | Ethiopia | Community based cross sectional | Reproductive age women |
| Kandala N. et al (2019)(107) | Kenya | Demographic health survey | ears old girls |
| Théra T. et al (2015)(108) | Mali | Hospital based cross sectional | Childbirth women |
| Minsart F. et al (2014 )(109) | Djibouti | Hospital based | Childbirth women |
| Kandala B.et al (2015)(110) | Senegal | Multiple Indicator Cluster Survey (MICS) | Women |
| Kandala B.et al (2015)(110) | Senegal | Multiple Indicator Cluster Survey (MICS) | daughters |
| Kaplan A. et al (2013)(111) | Gambia | Hospital based cross sectional | Women visited antenatal care, deliveries, and post-natal |
| Sichone A. et al (2019)(112) | Tanzania | Facility cross sectional | Women in labor |
| Bright O. et al (2013)(113) | Sierra Leone | Demographic health survey | Reproductive age women |
| Bright O. et al (2018)(113) | Mali | Demographic health survey | Reproductive age women |
| Duna A. et al (2017) (114) | Egypt | Demographic health survey | Reproductive age women |
| Engelbert A. et al(2013)(115) | Ghana | Hospital based cross sectional | Women in labour |
| Edmund N. et al (2019)(116) | Nigeria | National survey | Daughters of reproductive age women |
| Alphones K. et al (2019)(117) | Benin | National survey | Reproductive age women |
| [Mekuanint W](https://www.researchgate.net/profile/Mekuanint-Simeneh-Workie?utm_content=businessCard&utm_source=publicationDetail&rgutm_meta1=AC%3A21150225).et al (2020)(118) | Ethiopia | Demographic health survey | Reproductive age women |
| Bjälkander O. et al (2013)(119) | Sierra Leone | Community based cross sectional | Reproductive age women |
| Mohamed A. et al (2022) (120) | Somalia | Hospital based study | Women visited our clinic for a medical check-up |
| [Talal A](https://www.researchgate.net/scientific-contributions/Talal-A-Abdel-Raheem-2139251812?_sg%5B0%5D=aLiwhE7QCfqPI3Gb8VyD_X3r9HyhRpgztPYJ15dz0W2QVSOAaiIhGRTo9NFbArHv0rtonFI.TRuKUsojW7luw7PRWmtImRXXKsaN6vACFmOhU-fkP9F6OwYrGl53XivqWBm4Vvsy379U5ikJdYbfDx-yl2JgLA.BmJ8Ryrw6MM_O9Kv1-nRvGoaCht2Bi7IzZRVN_HiUVRCrgdNmyA-BW9AiuyBbOGm46Yj0PFB8Kc-BajlrMVgCg&_sg%5B1%5D=fs4Rl7WcbdZNTLp-33m7AwGVuJd_o-3vIboNAOrLbWhrv2j0dk_9NfTJiJnPQCsABmXMNUE.uqxGrbsxlzxCxAc8ZG5gwvop2pzLuuBv8nYo61rc-Wgqx0Tjgsf9BOH9RMd2inQO7G1ag6j6RO1swCMTOlQuEw). et al (2018)(121) | Egypt | Community based cross sectional | Reproductive age women |
| Abdelmoneim K. et al (2017)(122) | Sudan | Hospital based cross sectional | women attending the outpatient clinic |
| Onuchukwu V. et al (2017)(123) | Nigeria | Hospital based cross sectional | Antenatal care attendants |
| Amal Y. et al (2019)(124) | Egypt | Community based cross sectional | Rural married women |
| Azeze G. et al (2020)(102) | Ethiopia | Demographic health survey | Demographic health survey |
| Eman S. et al (2017)(125) | Egypt | Community based cross sectional | Reproductive age women |
| Gedion A. et al (2021)(102) | Ethiopia | Demographic health survey | Reproductive age women |
| Gedion A. et al (2021)(102) | Ethiopia | Demographic health survey | daughters |
| Aderibigbe S. et al (2018)(126) | Nigeria | Community based cross sectional | Reproductive age women |
| Edmund O. et al (2019) (127) | Nigeria | Demographic health survey | Reproductive age women |

**Reference**

1. Sakeah E, Debpuur C, Oduro AR, Welaga P, Aborigo R, Sakeah JK, et al. Prevalence and factors associated with female genital mutilation among women of reproductive age in the Bawku municipality and Pusiga District of northern Ghana. BMC Women's Health. 2018;18(1):150.

2. Hussein MA, Adem A, Mohammed MA. Knowledge, attitude and practice of female genital mutilation among women in Jigjiga Town, Eastern Ethiopia. Gaziantep Med J. 2013;19(3):164-8.

3. Gajaa M, Wakgari N, Kebede Y, Derseh L. Prevalence and associated factors of circumcision among daughters of reproductive aged women in the Hababo Guduru District, Western Ethiopia: a cross-sectional study. BMC Womens Health. 2016;16:42.

4. Anjulo B, Lambebo A. Prevalence and associated factors of Female genital mutilation among reproductive age women’s of Damot Gale woreda, Wolaita Zone, Southern Ethiopia: a cross-sectional study, 20182021.

5. Bogale D, Markos D, Kaso M. Prevalence of female genital mutilation and its effect on women's health in Bale zone, Ethiopia: a cross-sectional study. BMC Public Health. 2014;14:1076.

6. Obijiofor N, Enete C, Nnonyelu C, Umeononihu O, Emeka E, Okoro C, et al. Female Genital Mutilation: Prevalence, Awareness and Attitude among Igbo Women of Child-Bearing Age in Nigeria. Obstetrics and Gynecology Research. 2020;3:145-60.

7. Setegn T, Lakew Y, Deribe K. Geographic Variation and Factors Associated with Female Genital Mutilation among Reproductive Age Women in Ethiopia: A National Population Based Survey. PLOS ONE. 2016;11(1):e0145329.

8. Elbendary RN, Shokry DA, Deeb WS, Morsi EM. Female genital mutilation (FGM): Is it still an existing problem in Egypt? Forensic Sci Int. 2021;318:110574.

9. Tamire M, Molla M. Prevalence and belief in the continuation of female genital cutting among high school girls: a cross - sectional study in Hadiya zone, Southern Ethiopia. BMC Public Health. 2013;13:1120.

10. Gebrekirstos K, Abebe M, Fantahun A. A cross sectional study on factors associated with harmful traditional practices among children less than 5 years in Axum town, north Ethiopia, 2013. Reprod Health. 2014;11:46.

11. Desalegn S, Negussie D, Gamachu F, Tadele K, Markos D. Prevalence and associated factors of female genital mutilation among high school students in Dale Wabera Woreda, Oromia Regional State, Ethiopia. International Journal of Medicine and Medical Sciences. 2017;9(6):72-8.

12. Ashimi AO, Amole TG, Iliyasu Z. Prevalence and predictors of female genital mutilation among infants in a semi urban community in northern Nigeria. Sex Reprod Healthc. 2015;6(4):243-8.

13. Abdisa B, Beyene M, Tasew A. Assessment of the Prevalence of FGM and Associated Factors among Women’s of Reproductive Age Group in Kebirbeyah Town, Somali Region Eastern Ethiopia, 2017. Health Science Journal. 2017;11.

14. Eguvbe A, Okeoghene, Alabrah P, Waibode, Allagoa D, Oju, et al. European Journal of Public Health Studies AWARENESS, PREVALENCE AND PRACTICE OF FEMALE GENITAL MUTILATION AMONGST STUDENTS IN A TERTIARY INSTITUTION IN SOUTH-SOUTH NIGERIA AWARENESS, PREVALENCE AND PRACTICE OF FEMALE GENITAL MUTILATION AMONGST STUDENTS IN A TERTIARY INSTITUTION IN SOUTH-SOUTH NIGERIA. European Journal of Public Health Studies. 2022;5.

15. Ojeleye O, Ndikom C, Fa O. Perception and Practice of Female Genital Cutting among Mothers in Ibadan, Nigeria. International Journal of Nursing and Health Science. 2017;4:71-80.

16. Helamo D, Katama S, Leta T, Hibstu D. Prevalence of Female Genital Mutilation and its Association with Birth Complications among Women Attending Delivery Service in Nigist Eleni Mohammed General Hospital, Hossana, Southern Nations, Nationalities and Peoples' Region, Ethiopia. Reproductive System & Sexual Disorders. 2017;06.

17. Warsame M, Abdi O, Ali O. Assessment of Knowledge, Attitude and Practice Towards Female Genital cutting (FGC) among Women of Reproductive Age Group in Jigjiga City, Somali Region; Ethiopia: Community Based Cross-Sectional Study. 2021;4:1-5.

18. Abebe S, Dessalegn M, Hailu Y, Makonnen M. Prevalence and Barriers to Ending Female Genital Cutting: The Case of Afar and Amhara Regions of Ethiopia. Int J Environ Res Public Health. 2020;17(21).

19. Moges N, Kassa G. Knowledge, Attitude and Practice of Women Towards Female Genital Mutilation in Lejet Kebele, Dembecha Woreda, Amhara Regional State, Northwest, Ethiopia, 2014. Journal of Gynecology and Obstetrics. 2015;3.

20. Gebremariam K, Assefa D, Weldegebreal F. Prevalence and associated factors of female genital cutting among young adult females in Jigjiga district, eastern Ethiopia: a cross-sectional mixed study. International Journal of Women's Health. 2016;8:357-65.

21. Adıgüzel C, Bas Y, Erhan Mehmet D, Gelle Maimuna A. The Female Genital Mutilation/Cutting Experience in Somali Women: Their Wishes, Knowledge and Attitude. Gynecologic and Obstetric Investigation. 2018;84:1-10.

22. Omaka A, Obande-Ogbuinya E, Aleke Ph.D C, Eunice A, Nwafor J, Nwankwo O, et al. Demographic Predictors of Cultural Practices Regarding Female Genital Mutilation among Married Women in Ebonyi State, Nigeria. Journal of Advances in Medicine and Medical Research. 2021:23-31.

23. Gudu W, Abdulahi M. Labor, Delivery and Postpartum Complications in Nulliparous Women with Female Genital Mutilation Admitted to Karamara Hospital. Ethiop Med J. 2017;55(1):11-7.

24. Belda S, Tololu A. Knowledge, attitude and practice of mothers towards female genital mutilation in south west Shoa zone, Oromia region, Ethiopia. MOJ Public Health. 2017;6(2):279-86.

25. Jeremiah I. The Pattern of Female Genital Mutilation in Port Harcourt, Southern Nigeria. International Journal of TROPICAL DISEASE & Health. 2014;4:469-76.

26. Suleiman IR, Maro E, Shayo BC, Alloyce JP, Masenga G, Mahande MJ, et al. Trend in female genital mutilation and its associated adverse birth outcomes: A 10-year retrospective birth registry study in Northern Tanzania. PLOS ONE. 2021;16(1):e0244888.

27. Yasein YA. Prevalence of female genital mutilation among school girls in El- Mansoura Center, El-Dakahlia Governorate, Egypt. IOSR Journal of Dental and Medical Sciences. 2014;13:76-83.

28. Adewole A, Adayonfo EO, editors. PREVALENCE AND COMPLICATIONS OF FEMALE GENITAL MUTILATION AMONG PATIENTS AT THE UNIVERSITY OF BENIN TEACHING HOSPITAL, BENIN CITY2017.

29. Shawky M, Wahdan I, El-Nimr N. Prevalence of Female Genital Mutilation, and Women’s Knowledge, Attitude, and Intention to Practice in Egypt: A Nationwide Survey. Journal of High Institute of Public Health. 2020;50:139-45.

30. Anmut W, Toru T, Yeshambel A, Mesele M. Knowledge, Attitude and Practice Towards Female Genital Mutilation Among Reproductive Age Women in Amad Imam Town, Jarso District, East Hararge Zone, Oromia Region , Ethiopia: A Community Based Study. 2020:2020.

31. Balta A, Fanta L. Prevalence and associated factors of Female Genital Mutilation among reproductive age women’s of Wolayita Zone, Southern Ethiopia: A cross-sectional study. International Journal of Sexual and Reproductive Health Care. 2021:091-8.

32. Obi A, Ol I. Prevalence of Female Genital Mutilation and its Determinants among Pregnant Women in Benin City, Nigeria. 2018;30:12-21.

33. Muchene K, Mageto I, Jebet J. Knowledge and Attitude on Obstetric Effects of Female Genital Mutilation among Maasai Women in Maternity Ward at Loitokitok Sub-County Hospital, Kenya. Obstetrics and Gynecology International. 2018;2018:1-5.

34. Greis A, Bärnighausen T, Bountogo M, Ouermi L, Sié A, Harling G. Attitudes towards female genital cutting among adolescents in rural Burkina Faso: a multilevel analysis. Trop Med Int Health. 2020;25(1):119-31.

35. Abolfotouh SM, Ebrahim AZ, Abolfotouh MA. Awareness and predictors of female genital mutilation/cutting among young health advocates. Int J Womens Health. 2015;7:259-69.

36. Alemu AA. Trends and Determinants of Female Genital Mutilation in Ethiopia: Multilevel Analysis of 2000, 2005 and 2016 Ethiopian Demographic and Health Surveys. Int J Womens Health. 2021;13:19-29.

37. CSA I. Ethiopia demographic and health survey 2016: key indicators report. Central statistics agency (CSA)[Ethiopia] and ICF Addis Ababa, and Rockville: CSA and ICF. 2016.

38. Elduma AH. Female Genital Mutilation in Sudan. Open Access Maced J Med Sci. 2018;6(2):430-4.

39. Gudeta TA, Regassa TM, Gamtessa LC. Female genital mutilation: prevalence, associated factors and health consequences among reproductive age group women in Keffa Zone, Southwest, Ethiopia. Reproductive Health. 2022;19(1):60.

40. Ahinkorah BO. Factors associated with female genital mutilation among women of reproductive age and girls aged 0-14 in Chad: a mixed-effects multilevel analysis of the 2014-2015 Chad demographic and health survey data. BMC Public Health. 2021;21(1):286.

41. Tesema G, Teshale A, Agegnehu C, Alem A, Liyew A, Yeshaw Y, et al. Trends and Spatial Variation of Female Genital Mutilation among Reproductive Age Women in Ethiopia based on 2000, 2005, and 2016 Ethiopian Demographic and Health Surveys: Spatial-temporal and Multivariate Decomposition Analysis2019.

42. Alhassan AR, Anyinzaam-Adolipore JN. Female Genital Mutilation in Ghana: Prevalence and Socioeconomic Predictors. Biomed Res Int. 2021;2021:6675579.

43. Melese G, Tesfa M, Sharew Y, Mehare T. Knowledge, attitude, practice, and predictors of female genital mutilation in Degadamot district, Amhara regional state, Northwest Ethiopia, 2018. BMC Women's Health. 2020;20(1):178.

44. Mbogo B, Karanja S, Omwaka K, Lugayo D, Leshore C. Underlying Sociocultural Practices Influencing Prevalence of Female Genital Mutilation/Cutting in Kajiado County. Advances in Sexual Medicine. 2019;09:17-28.

45. Elnimeiri M, Abdelbasit R, Ibrahim M, Mingaryous D, Abdelrahim T, Satti S. Determinants of Female Genital Mutilation/Cutting in Khartoum State - Sudan, 2020: A Cross-Sectional Study2020.

46. Birge Ö, Serin AN, Bakır MS. Female genital mutilation/cutting in sudan and subsequent pelvic floor dysfunction. BMC Women's Health. 2021;21(1):430.

47. Yasein Y. Prevalence of female genital mutilation among school girls in ElMansoura Center, El-Dakahlia Governorate, Egypt. IOSR Journal of Dental and Medical Sciences. 2014;13:76-83.

48. Chinawa A, Chinawa JM, Ossai EN, Aronu A, Ozokoli GE, Enebe JT. Pattern of Female Genital Mutilation among Adolescents Attending Secondary Schools' in Enugu Metropolis. Journal of tropical pediatrics. 2020.

49. Ahmed MR, Shaaban MM, Meky HK, Amin Arafa ME, Mohamed TY, Gharib WF, et al. Psychological impact of female genital mutilation among adolescent Egyptian girls: a cross-sectional study. Eur J Contracept Reprod Health Care. 2017;22(4):280-5.

50. Yismaw AE, Tadesse Z, Gessesse DN, Tarekegn AA, Yismaw YE. Spatial distribution and associated factors of female genital cutting among reproductive-age women in Ethiopia: Further analysis of EDHS 2016. Clinical Epidemiology and Global Health. 2021;12:100858.

51. Shaheen H, Kasemy Z, Salah F. The current situation regarding awareness about female genital mutilation among men working in schools of Benha City, Qaluobia Governorate2017.

52. Ibrahim B, Ahmed Z, Ado A, Mohammed Y, Abubakar A, Balogun M, et al. Prevalence and Determinants of Female Genital Mutilation among Women in a Rural Settlement of Kano State Nigeria, 2016. Merit Research Journal of Medicine and Medical Sciences. 2017;5:072-7.

53. Grose RG, Hayford SR, Cheong YF, Garver S, Kandala NB, Yount KM. Community Influences on Female Genital Mutilation/Cutting in Kenya: Norms, Opportunities, and Ethnic Diversity. J Health Soc Behav. 2019;60(1):84-100.

54. Anyanwu CE, Torpey K, Abiodun OP, Sanni OF, Anyanwu ID. Variations in the Prevalence of Female Genital Mutilation Among Reproductive-aged Women in Nigeria Across Three Generations. Int J MCH AIDS. 2022;11(2):e548.

55. Ojo TO, Ijadunola MY. Sociodemographic factors associated with female genital cutting among women of reproductive age in Nigeria. Eur J Contracept Reprod Health Care. 2017;22(4):274-9.

56. Osuorah C. Sociodemographic Predictors of Genital Mutilation (Circumcision) of the Girl Child in Nigeria: A Population-Based Study. International Journal of Women's Health and Reproduction Sciences. 2015;3:142-50.

57. Setegn M. Community Participation and Challenges in controlling Female Genital Mutilation in A Rural Community of Southwest Ethiopia: A Mixed Study2021.

58. Masho SW, Orekoya O, Lowery E, Wallenborn JT. Female genital mutilation and contraceptive use: findings from the 2014 Egypt demographic health survey. Int J Public Health. 2020;65(7):1151-8.

59. Abolfotouh S EA, Abolfotouh M. Awareness and predictors of female genital mutilation/cutting among young health advocates. Int J Womens Health. 2015;7:259-269

<https://doi.org/10.2147/IJWH.S78664>

2105.

60. Idung A, Okokon I. Beliefs and Prevalence of Female Genital Circumcision among Pregnant Women Attending Ante-natal Clinic in a Mission Hospital in Uyo, Akwa Ibom State, Nigeria. British Journal of Medicine and Medical Research. 2017;19:1-8.

61. Alkhalaileh D, Hayford SR, Norris AH, Gallo MF. Prevalence and attitudes on female genital mutilation/cutting in Egypt since criminalisation in 2008. Cult Health Sex. 2018;20(2):173-82.

62. Gebrekirstos K, Fantahun A, Buruh G. Magnitude and Reasons for Harmful Traditional Practices among Children Less Than 5 Years of Age in Axum Town, North Ethiopia, 2013. Int J Pediatr. 2014;2014:169795-.

63. Mohammed AY ET, Wodera AL. Assessment of Knowledge, Attitude and Practice towards Female Genital Mutilation Among community of Agarfa Town, Southeast Ethiopia. J Comm Pub Health Nursing 6: 273. DOI: 10.4172/2471-9846.1000273

2021.

64. Sharfi AR, Elmegboul MA, Abdella AA. The continuing challenge of female genital mutilation in Sudan. African Journal of Urology. 2013;19(3):136-40.

65. Alhassan AR, Anyinzaam-Adolipore J. Female Genital Mutilation in Ghana: Prevalence and Socioeconomic Predictors. BioMed Research International. 2021;2021:1-6.

66. Besera G, Roess A. The relationship between female genital cutting and women's autonomy in Eritrea. Int J Gynaecol Obstet. 2014;126(3):235-9.

67. Galukande M, Kamara J, Ndabwire V, Leistey E, Valla C, Luboga S. Eradicating female genital mutilation and cutting in Tanzania: an observational study. BMC Public Health. 2015;15:1147.

68. Kalu A, Belda S. Knowledge, Attitude and Practice of Mothers towards Female Genital Mutilation in South West Shoa Zone, Oromia Region, Ethiopia. MOJ Public Health. 2017;6.

69. Lamiso Y, Borsamo A, Abeje S. What are the Complications Associated with Female Genital Mutilation Among Postnatal Women in Chuko Primary Hospital, Sidama Regional State, Ethiopia?2021.

70. Ouldzeidoune N, Keating J, Bertrand J, Rice J. A Description of Female Genital Mutilation and Force-Feeding Practices in Mauritania: Implications for the Protection of Child Rights and Health. PloS one. 2013;8:e60594.

71. Kandala N-B, Komba P. Geographic Variation of Female Genital Mutilation and Legal Enforcement in Sub-Saharan Africa: A Case Study of Senegal. The American journal of tropical medicine and hygiene. 2015;92.

72. Yaya S, Ghose B. Female Genital Mutilation in Nigeria: A Persisting Challenge for Women’s Rights. Social Sciences. 2018;7(12):244.

73. Okunade K, Okunowo A, Omisakin S, Ajepe G. An institutional survey of female genital mutilation in Lagos, South-West, Nigeria Author for Correspondence. Orient Journal of Medicine. 2016;28:28-35.

74. Andualem M. DETERMINANTS OF FEMALE GENITAL MUTILATION PRACTICES IN EAST GOJJAM ZONE, WESTERN AMHARA, ETHIOPIA. Ethiop Med J. 2016;54(3):109-16.

75. Mohammed ES, Seedhom AE, Mahfouz EM. Female genital mutilation: current awareness, believes and future intention in rural Egypt. Reprod Health. 2018;15(1):175.

76. Chinawa AT, Chinawa JM, Ossai EN, Aronu AE, Ozokoli GE, Enebe J. Pattern of Female Genital Mutilation among Adolescents Attending Secondary Schools' in Enugu Metropolis. J Trop Pediatr. 2021;67(1).

77. Gbadebo BM, Salawu AT, Afolabi RF, Salawu MM, Fagbamigbe AF, Adebowale AS. Cohort analysis of the state of female genital cutting in Nigeria: prevalence, daughter circumcision and attitude towards its discontinuation. BMC Womens Health. 2021;21(1):182.

78. Oljira T, Assefa N, Dessie Y. Female genital mutilation among mothers and daughters in Harar, eastern Ethiopia. International Journal of Gynecology & Obstetrics. 2016;135(3):304-9.

79. Tamire M, Molla M. Prevalence and belief in the continuation of female genital cutting among high school girls: a cross-sectional study in Hadiya zone, Southern Ethiopia. BMC public health. 2013;13(1):1-9.

80. Gebremariam K, Assefa D, Weldegebreal F. Prevalence and associated factors of female genital cutting among young adult females in Jigjiga district, eastern Ethiopia: a cross-sectional mixed study. Int J Womens Health. 2016;8:357.

81. Shiferaw D, Deyessa N, Kinati T, Fufa G, Beyene M. Prevalence and associated factors of female genital mutilation among high school students in Dale Wabera Woreda , Oromia Regional State, Ethiopia. international journal of medicine and medical science. 2017;9.

82. Andualem M. Female genital mutilation and associated factors in GonchaSiso-enessie district, east gojjam zone, Amhara region, Ethiopia (2012). J Health Med Informat. 2013;4(142):2.

83. Nurilign Abebe Moges GM, Mihiretie Gedfew, Mohammednur Redi, Mohammed Molla, Setarg Ayenew, Shegaw Fentahun, Solomon Adisie, Zewudu Dagnew. Knowledge, Attitude and Practice of Women Towards Female Genital Mutilation in Lejet Kebele, Dembecha Woreda, Amhara Regional State, Northwest, Ethiopia. Journal of Gynecology and Obstetrics. Vol. 3, No. 2, 2015, pp. 21-25. doi: 10.11648/j.jgo.20150302.11.

84. Gebrekidan K, Abebe M, Aregay A. A cross sectional study on factors associated with harmful traditional practices among children less than 5 years in Axum town, north Ethiopia, 2013. Reproductive health. 2014;11:46.

85. Anjulo B, Lambebo A. Prevalence and associated factors of Female genital mutilation among reproductive age women’s of Wolayita Zone, Southern Ethiopia: a cross-sectional study, 20182021.

86. Alhassan AR, Anyinzaam-Adolipore JN. Female Genital Mutilation in Ghana: Prevalence and Socioeconomic Predictors. BioMed Research International. 2021;2021:6675579.

87. Mboge B, Knapp KA, Tantsyura V, Jagne SF, Alamgir H. Female genital cutting in the Gambia: can education of women bring change? Journal of public health. 2019.

88. Ejigu Y, Tiruneh G, Mekonnen M, Kibret GD. Prevalence and Contributing Factors of Female Genital Cutting in Debaytilatgin District, Northwest Ethiopia. clinics in Mother and Child Health. 2014;11:1-6.

89. Mbogo B, Karanja, S. , Omwaka, K. , Lugayo, D. and Leshore, C. . Underlying Sociocultural Practices Influencing Prevalence of Female Genital Mutilation/Cutting in Kajiado County. Advances in Sexual Medicine, 9, 17-28. doi: 10.4236/asm.2019.92002. 2019.

90. B Obijiofor N, A Enete C, E Nnonyelu C, S Umeononihu O, A Emeka E, C Okoro C, et al., editors. Female Genital Mutilation: Prevalence, Awareness and Attitude among Igbo Women of Child-Bearing Age in Nigeria2020.

91. Chuluko BG, Abeya SG, editors. Female Genital Mutilation/Cutting and the Occurrences of Birth Complications Among Women of Reproductive Age in Gewane Woreda, Afar Regional State, Ethiopia2018.

92. Fosu M, Nyarko IPR, Anokye M. Female Genital Mutilation/Cutting among Ghanaian Women: The Determinants. Research on humanities and social sciences. 2014;4:61-8.

93. Ahmed MR, Shaaban MM, Meky HK, Amin Arafa ME, Mohamed TY, Gharib WF, et al. Psychological impact of female genital mutilation among adolescent Egyptian girls: a cross-sectional study. The European Journal of Contraception & Reproductive Health Care. 2017;22:280 - 5.

94. Ibrahim BS, Ahmed ZD, Ado A, Mohammed Y, Abubakar AA, Balogun MS, et al., editors. Prevalence and Determinants of Female Genital Mutilation among Women in a Rural Settlement of Kano State Nigeria , 20162017.

95. Shaheen HM, Kasemy ZA, Eldeen FMS. The current situation regarding awareness about female genital mutilation among men working in schools of Benha City, Qaluobia Governorate. Menoufia Medical Journal. 2017;30(1):23.

96. Tesema G, Teshale A, Alem A, Liyew A, Yeshaw Y, Kebede S. Trends and Spatial Variation of Female Genital Mutilation among Reproductive Age Women in Ethiopia based on 2000, 2005, and 2016 Ethiopian Demographic and Health Surveys: Spatial-temporal and Multivariate Decomposition Analysis2019.

97. Yassin K, Idris HA, Ali AA. Characteristics of female sexual dysfunctions and obstetric complications related to female genital mutilation in Omdurman maternity hospital, Sudan. Reproductive Health. 2018;15(1):7.

98. Ahmed M, Seid A, Seid S, Yimer A. Does previous circumcision and wealth index influence women’s attitude to discontinue the practice of female genital mutilation and cutting (FGM/C) in Ethiopia? PLOS ONE. 2022;17(8):e0272934.

99. Olabisi AA, Olumuyiwa AJ, Saidat IB. Awareness and practice of female genital mutilation/cutting in a semi-urban community in southwest Nigeria. Asian Journal of Medical and Biological Research. 2019.

100. Sougou N, Seck I. Female genital mutilation remains high in Senegal: an analysis of DHS 2018. European Journal of Public Health. 2021;31.

101. Danyah S. Factors associated with Female Genital Mutilation among daughters of reproductive aged women at Academic Charity Teaching Hospital (ACTH). Clinical Journal of Obstetrics and Gynecology. 2021;4:073-80.

102. Azeze GA, Williams A, Tweya H, Obsa MS, Mokonnon TM, Kanche ZZ, et al. Changing prevalence and factors associated with female genital mutilation in Ethiopia: Data from the 2000, 2005 and 2016 national demographic health surveys. PLoS One. 2020;15(9):e0238495.

103. Inungu JN, Tou Y. Factors associated with female genital mutilation in Burkina Faso. Journal of public health and epidemiology. 2013;5:20-8.

104. Geremew TT, Azage M, Mengesha EW. Hotspots of female genital mutilation/cutting and associated factors among girls in Ethiopia: a spatial and multilevel analysis. BMC Public Health. 2021;21.

105. Akpan B, Idung E. Female Genital Mutilation among Antenatal Attendees at St. Luke’s Hospital Anua, Uyo, Nigeria. South Asian Research Journal of Nursing and Healthcare. 2019;01:62-8.

106. Anmut W. Knowledge, Attitude and Practice Towards Female Genital Mutilation Among Reproductive Age Women in Amad Imam Town, Jarso District, East Hararge Zone, Oromia Region , Ethiopia: A Community Based Study. Journal of Medicine, Physiology and Biophysics. 2020;66:14-21.

107. Kandala NB, Nnanatu CC, Atilola G, Komba P, Mavatikua L, Moore Z, et al. A Spatial Analysis of the Prevalence of Female Genital Mutilation/Cutting among 0-14-Year-Old Girls in Kenya. Int J Environ Res Public Health. 2019;16(21).

108. Théra T, Kouma A, Touré M, Coulibaly A, Sima M, Ongoiba I, et al. [Obstetrical complications of genital mutilation in Malian rural environment]. J Gynecol Obstet Biol Reprod (Paris). 2015;44(3):276-9.

109. Minsart AF, N'Guyen T S, Ali Hadji R, Caillet M. Maternal infibulation and obstetrical outcome in Djibouti. J Matern Fetal Neonatal Med. 2015;28(14):1741-6.

110. Kandala N-B, Komba PN. Geographic variation of female genital mutilation and legal enforcement in sub-Saharan Africa: a case study of Senegal. Am J Trop Med Hyg. 2015;92(4):838-47.

111. Kaplan A, Forbes M, Bonhoure I, Utzet M, Martín M, Manneh M, et al. Female genital mutilation/cutting in The Gambia: long-term health consequences and complications during delivery and for the newborn. Int J Womens Health. 2013;5:323-31.

112. Sichone et al TeoFGMopiawiliDR, Tanzania. South Sudan Medical Journal 2019; 12(4) 121-123.

113. Ahinkorah BO, Hagan JE, Seidu AA, Budu E, Armah-Ansah EK, Adu C, et al. Empirical linkages between female genital mutilation and multiple sexual partnership: evidence from the 2018 Mali and 2013 Sierra Leone Demographic and Health Surveys. J Biosoc Sci. 2021:1-16.

114. Alkhalaileh D, Hayford S, Norris A, Gallo M. Prevalence and attitudes on female genital mutilation/cutting in Egypt since criminalisation in 2008. Culture, Health & Sexuality. 2017;20:1-10.

115. Nonterah E, Kanmiki E, Agorinya I, Sakeah E, Tamimu M, Kagura J, et al. Prevalence and adverse obstetric outcomes of female genital mutilation among women in rural Northern Ghana. The European Journal of Public Health. 2019;ckz195.

116. Aniwada E. Prevalence and Predictors of Female Genital Cutting in Nigeria: an Analysis of 2013 Nigeria Demographic and Health Survey. 2018.

117. Kpozehouen A, Glèlè Ahanhanzo Y, Klikpo E, Azandjeme C, Biaou C, Sossa C, et al. Female Genital Mutilation in Benin: Prevalence and Associated Factors Based on Data from the Demographic and Health Survey. World Journal of Public Health. 2019;4.

118. Workie MS. Bayesian Binary Logistic Generalized Linear Mixed Models of Female Genital Mutilation2020.

119. Bjälkander O, Grant DS, Berggren V, Bathija H, Almroth L. Female genital mutilation in sierra leone: forms, reliability of reported status, and accuracy of related demographic and health survey questions. Obstet Gynecol Int. 2013;2013:680926.

120. Mohamed AH, Mohamud RYH, Mohamud HA, Eraslan A, Gur M, Omar AA, et al. Somalian women with female genital mutilation had increased risk of female sexual dysfunction: a cross-sectional observational study. Sci Rep. 2022;12(1):15633.

121. Raheem T, El-tahalawi S, Raia N, Elsary A, Ibrahem K. The effect of female genital mutilation on couple sexual function. International Journal Of Community Medicine And Public Health. 2018;5:905.

122. Kheir A. THE IMPACT OF FEMALE GENITAL MUTILATION ON THE SEXUAL FUNCTION OF WOMEN ATTENDING THE ACADEMY TEACHING HOSPITAL IN SUDAN. Journal of Disease and Global Health. 2017;10:1-6.

123. Uchenna O, Okwuchukwu O, Nwafor J, Joy A, Chukwu I, Blessing O, et al. Female Genital Mutilation among Antenatal Clinic Attendees at Alex Ekwueme Federal University Teaching Hospital, Abakaliki. 2019:39-43.

124. Elwahed A, Ali W, Nicola V. Expereince of Female Genital Cutting and Sexual Satisfaction Among Rural Married Women in El Beheria Goveronorate. International Journal of Studies in Nursing. 2019;4:46.

125. Mohammed ES, Seedhom AE, Mahfouz EM. Female genital mutilation: current awareness, believes and future intention in rural Egypt. Reproductive Health. 2018;15(1):175.

126. Aderibigbe S, Ameen A-M, Salaudeen AG, Mohammed Jimoh S, Uthman MM, Bolarinwa O, et al. Practice of Female Genital Cutting Amonst Adults in Ilorin Metropolis North Central Nigeria. 2018;25.

127. Ossai E, Aniwada E, Ibiok N, Nwobi E. Maternal Factors Associated with Female Genital Cutting of Daughters in Nigeria: A National Population Based Study. 2019.
