# Supplementary material for: Prevalence of female genital mutilation and associated factors among women and girls in Africa: a systematic review and meta-analysis
Source: Syst Rev. 2024 Jan 12;13:26. doi: 10.1186/s13643-023-02428-6 (PMC10785359; doi:10.1186/s13643-023-02428-6)
Supplement: Supplementary file 3 — Additional file 3: Table 1. Risk of bias assessment for cross-sectional studies – Newcastle-Ottawa Scale (adaptation). Table 2. Risk of bias assessment for cohort studies – Newcastle-Ottawa Scale (adaptation). [file 13643_2023_2428_MOESM3_ESM.docx]

**Additional file 2**

Table 1: Risk of bias assessment for cross-sectional studies – Newcastle-Ottawa Scale (adaptation)

| **NOS scale for cross-sectional studies** | Selection | Comparability | Outcome | Total score |  |  |  |  |
| --- | --- | --- | --- | --- | --- | --- | --- | --- |
|  | Representativeness  (1) | Sample size  (1) | Non-respondents  (1) | Ascertainment of the exposure (risk factor)    (2) | The subjects in different outcome groups are comparable, based on the study design or analysis. Confounding factors are controlled  (2) | Assessment of the outcome  (2) | Statistical test  (1) | **Total score** |
| Sakeah, E. et al(2018) (1) | * | * | * | ** | * | ** | * | 9 |
| Hussein M. et al (2013)(2) | * | * | * | * | ** | ** | * | 9 |
| Gajja M. et al (2016) (3) | * | * | * | ** | * | ** | * | 9 |
| Gajja M. et al (2016) (3) | * | * | * | * | * | ** | * | 8 |
| Bargude B. et al (2018)(4) | * | * | * | ** | * | ** | * | 9 |
| Bogale, D. et al (2014) (5) | * | * | * | ** | * | ** | * | 9 |
| Bogale, D. et al (2014)(5) | * | * | * | ** | * | ** | * | 9 |
| Nkechi B. et al (2020)(6) | * | * | * | * | * | ** | * | 8 |
| Setegni M. et al(2016)(7) | * | * | * | ** | * | ** | * | 9 |
| Setegni M. et al(2016)(7) | * | * | * | * | * | ** | * | 9 |
| Elbendary R. et al (2021)(8) | * | * | * | ** | * | ** | * | 8 |
| Tamire M. et al (2013)(9) | * | * | * | ** | * | ** | * | 9 |
| Gebrekirstos K. et al (2013)(10) | * | * | * | ** | * | ** | * | 9 |
| Desalegn S. et al(2017)(11) | * | * | * | ** | * | ** | * | 9 |
| Ashimi A. et al (2017)(12) | * | * | * | * | ** | ** | * | 9 |
| Bayis B. et al (2017)(13) | * | * | * | ** | * | ** | * | 9 |
| Anthony A. et al (2022)(14) | * | * | * | * | * | ** | * | 8 |
| Chizoma M. et al (2017)(15) | * | * | * | ** | * | ** | * | 9 |
| Degefa H. et al (2017)(16) | * | * | * | ** | * | ** | * | 9 |
| Muktar S. et al (2021)(17) | * | * | * | ** | * | ** | * | 9 |
| Abebe S. et al (2020)(18) | * | * | * | * | * | * | * | 7 |
| Abebe S. et al (2020)(18) | * | * | * | ** | * | ** | * | 9 |
| Nurilign A. et al(2015)(19) | * | * | * | * | * | ** | * | 9 |
| Nurilign A. et al(2015)(19) | * | * | * | ** | * | ** | * | 8 |
| Kidanu G. et al (2022)(20) | * | * | * | ** | * | ** | * | 9 |
| Cevdet A. et al (2017) (21) | * | * | * | ** | * | ** | * | 9 |
| Amari O. et al (2021) (22) | * | * | * | ** | * | ** | * | 9 |
| Wondimu G. et al (2017)(23) | * | * | * | * | ** | ** | * | 9 |
| Belda S. et al (2017)(24) | * | * | * | ** | * | ** | * | 9 |
| Israel J. et al (2014)(25) | * | * | * | * | * | ** | * | 8 |
| Suleiman I. et al (2014)(26) | * | * | * | ** | * | ** | * | 9 |
| Yasser A. et al (2014)(27) | * | * | * | ** | * | ** | * | 9 |
| Adewole A. et al (2017)(28) | * | * | * | ** | * | ** | * | 9 |
| Adewole A. et al (2017)(28) | * | * | * | * | * | ** | * | 8 |
| Marwa S. et al (2020)(29) | * | * | * | ** | * | ** | * | 9 |
| Walellign A. et al (2020)(30) | * | * | * | ** | * | ** | * | 9 |
| [Anjulo B](https://www.researchgate.net/scientific-contributions/Anjulo-Bargude-Balta-2206363533?_sg%5B0%5D=oKhNGiqN9Y60yevIYGYMluwehmEl7vLWLwHqdjhomkGBtYmtZDVVMfKIhrX-cZ2KKzkoFw8._6Cs6Zbk3AHmFDdZdUcN1miJ7jBj0xkX1IEXSoo28THIxRTchvN0N0cbywDnRCvqv4uP8LkEUDxL7jY9pu9oKg&_sg%5B1%5D=aocOZHl-_26bszLYuD1Jm1K5pUi0b2pwUyJ8UudooZNtHalQAwAz8uZhbob0Ji7V6FkXUM4.1vW_ckdEGnJr4UwXjKLl42dM9AbPaC6tfUpd9hWY8WQ0gfVtKV6NkgfhJuyV7I2QHLMujIgpfhN506-LJIjrAw). et al (2021)(31) | * | * | * | ** | * | ** | * | 9 |
| Obi A. et al (2018)(32) | * | * | * | ** | * | ** | * | 9 |
| Obi A. et al (2018)(32) | * | * | * | ** | * | ** | * | 9 |
| Keddy W. et al (2017)(33) | * | * | * | ** | * | ** | * | 9 |
| Greis A. et al (2020)(34) | * | * | * | * | * | * | * | 7 |
| Abolfotouh S. et al (2015 )(35) | * | * | * | ** | * | ** | * | 9 |
| Alemu A.et al (2021)(36) | * | * | * | * | * | ** | * | 8 |
| Alemu A.et al (2021)(36) | * | * | * | ** | * | ** | * | 9 |
| Chinawa A. et al (2021)(36) | * | * | * | ** | * | ** | * | 9 |
| EDHS (2016)(37) | * | * | * | ** | * | ** | * | 9 |
| Adel H. et al (2018)(38) | * | * | * | * | * | ** | * | 8 |
| Adel H. et al (2018)(38) | * | * | * | ** | * | ** | * | 9 |
| Tesfaye A. et al (2022)(39) | * | * | * | * | * | ** | * | 9 |
| Tesfaye A. et al (2022) (39) | * | * | * | ** | * | ** | * | 8 |
| Ahinkorah B. et al (2021)(40) | * | * | * | ** | * | ** | * | 9 |
| Tesema G. et al (2020)(41) | * | * | * | ** | * | ** | * | 9 |
| Alhassan A. et al (2021)(42) | * | * | * | ** | * | ** | * | 9 |
| Melese G. et al (2018)(43) | * | * | * | * | ** | ** | * | 9 |
| [Bernard M](https://www.researchgate.net/scientific-contributions/Bernard-Mbogo-2156379663?_sg%5B0%5D=rJsd2SbucMQHtWiESPhwjcwiAdluETZnF1kG-1bNddFlHQtEyfaWiYu-A-klFzX9QnSUsp4.RkaMqHrCoOQhAlfrL0tm1RUI0pxT5mlJpIEFpSmtU8xv2nEbHq9UclCuXkAD6jrV39xfEmQCQNJXobEiKBBFWg&_sg%5B1%5D=7usygW96NpWMOtfpO7tHa2iytbCik-ICPsRRABknVJNWEHGhTpi87-oFR_j_JGvYoB1U6r0.YD__KwApvtiIdgCLzM6_jFeU3star63FSA5vat7EG0wcB-oc_-wzyvczPbUOHVYQNPWa4igBekWxGanIUvqPCw). et al (2019) (44) | * | * | * | ** | * | ** | * | 9 |
| Mustafa K. et al (2020)(45) | * | * | * | * | * | ** | * | 8 |
| Birge Ö. et al (2021)(46) | * | * | * | ** | * | ** | * | 9 |
| Yasser A. et al (2014)(47) | * | * | * | ** | * | ** | * | 9 |
| Awoere T. et al (2020)(48) | * | * | * | ** | * | ** | * | 9 |
| Ahmed M. et al (2017)(49) | * | * | * | * | * | ** | * | 8 |
| Ayenew A. et al (2021)(50) | * | * | * | ** | * | ** | * | 9 |
| Hala M. et al(2017)(51) | * | * | * | * | * | ** | * | 9 |
| Baffa S. et al (2016)(52) | * | * | * | ** | * | ** | * | 8 |
| Baffa S. et al (2016)(52) | * | * | * | ** | * | ** | * | 9 |
| Rose G. et al (2019)(53) | * | * | * | ** | * | ** | * | 9 |
| Rose G. et al (2019)(53) | * | * | * | ** | * | ** | * | 9 |
| Anyanwu C. et al (2021)(54) | * | * | * | * | ** | ** | * | 9 |
| [Ojo](https://pubmed.ncbi.nlm.nih.gov/?term=Ojo+TO&cauthor_id=28641026) T. et al (2017)(55) | * | * | * | ** | * | ** | * | 9 |
| Chideber I. et al(2015)(56) | * | * | * | * | * | ** | * | 8 |
| Achia T. et al (2014) | * | * | * | ** | * | ** | * | 9 |
| Melsew S. et al (2021)(57) | * | * | * | ** | * | ** | * | 9 |
| Masho W. et al (2014)(58) | * | * | * | ** | * | ** | * | 9 |
| Abolfotouh S. et al (2104)(59) | * | * | * | * | * | ** | * | 8 |
| Alphonsus I. et al (2017)(60) | * | * | * | ** | * | ** | * | 9 |
| Alkhalaileh D. et al (2018)(61) | * | * | * | * | * | ** | * | 9 |
| Gebre K. et al (2014)(62) | * | * | * | ** | * | ** | * | 8 |
| Ahmed Y. et al (2021)(63) | * | * | * | ** | * | ** | * | 9 |
| [Sharfi](https://www.sciencedirect.com/science/article/pii/S111057041300060X#!) [M](https://www.sciencedirect.com/science/article/pii/S111057041300060X#!). et al (2013)(64) | * | * | * | ** | * | ** | * | 9 |
| Abdul A. et al (2021)(65) | * | * | * | ** | * | ** | * | 9 |
| Besera G. et al (2014)(66) | * | * | * | * | ** | ** | * | 9 |
| Besera G. et al (2014)(66) | * | * | * | ** | * | ** | * | 9 |
| Mosess G. et al (2015)(67) | * | * | * | * | * | ** | * | 8 |
| Abdurehman K. et al (2017)(68) | * | * | * | ** | * | ** | * | 9 |
| Yirgalem Y. et al (2021)(69) | * | * | * | ** | * | * | * | 8 |
| Nacerdine O. et al (2013)(70) | * | * | * | ** | * | ** | * | 9 |
| Ngianga B. et al (2015)(71) | * | * | * | * | * | ** | * | 8 |
| Ngianga B. et al (2015)(71) | * | * | * | ** | * | ** | * | 9 |
| Yaya S. et al (2018)(72) | * | * | * | * | * | ** | * | 9 |
| Kehinde S. et al (2016)(73) | * | * | * | ** | * | ** | * | 8 |
| Yaya S. et al (2018)(72) | * | * | * | ** | * | ** | * | 9 |
| Andualem M. et al (2016)(74) | * | * | * | ** | * | ** | * | 9 |
| Andualem M. et al (2016)(74) | * | * | * | ** | * | ** | * | 9 |
| [Eman S](https://pubmed.ncbi.nlm.nih.gov/?term=Mohammed+ES&cauthor_id=30333019). et al (2018)(75) | * | * | * | * | ** | ** | * | 9 |
| Chinawa A. et al (2021)(76) | * | * | * | ** | * | ** | * | 9 |
| Gbadebo B. et al (2021)(77) | * | * | * | * | * | ** | * | 8 |
| Gbadebo B. et al (2021)(77) | * | * | * | ** | * | ** | * | 9 |
| Teshome O.et al (2016)(78) | * | * | * | ** | * | ** | * | 9 |
| Teshome O.et al (2016)(78) | * | * | * | ** | * | ** | * | 9 |
| Tamire M. et al (2013)(79) | * | * | * | * | * | ** | * | 8 |
| Gebremedhin K.et al (2016)(80) | * | * | * | ** | * | ** | * | 9 |
| Desalegn S. et al (2017)(81) | * | * | * | * | * | ** | * | 9 |
| Andualem M. et al (2013)(82) | * | * | * | ** | * | ** | * | 8 |
| Andualem M. et al (2013)(82) | * | * | * | ** | * | ** | * | 9 |
| Nurlign A. et al (2014)(83) | * | * | * | ** | * | ** | * | 9 |
| Gebrekirstos, K. et al (2014)(84) | * | * | * | ** | * | ** | * | 9 |
| Bargude B. et al (2021)(85) | * | * | * | * | ** | ** | * | 9 |
| Abdul R. et al(2021)(86) | * | * | * | * | * | ** | * | 9 |
| Baturu M. et al (2019)(87) | * | * | * | * | * | ** | * | 8 |
| Yaregal E.et al (2014)(88) | * | * | * | ** | * | ** | * | 9 |
| Bernand M. et al (2019)(89) | * | * | * | ** | * | ** | * | 9 |
| Nkechi O. et al (2020)(90) | * | * | * | ** | * | ** | * | 9 |
| Bayush G. et al (2018)(91) | * | * | * | * | * | ** | * | 8 |
| [Fosu](https://www.semanticscholar.org/author/M.-Fosu/52072294) M. et al (2014)(92) | * | * | * | * | * | ** | * | 9 |
| [Ahmed](https://www.semanticscholar.org/author/M.-R.-Ahmed/47285582), [M](https://www.semanticscholar.org/author/M.-M.-Shaaban/153353236). et al (2017)(93) | * | * | * | ** | * | ** | * | 8 |
| [Ibrahim](https://www.semanticscholar.org/author/B.-Ibrahim/16787231) B. et al (2016)(94) | * | * | * | ** | * | ** | * | 9 |
| [Shaheen](https://scholar.google.com/citations?user=TwmfE_sAAAAJ&hl=en&oi=sra) H. et al (2017)(95) | * | * | * | ** | * | ** | * | 9 |
| Getayeneh A. et al (2020)(96) | * | * | * | ** | * | ** | * | 9 |
| Ahmed M. et al (2022)(97) | * | * | * | * | ** | ** | * | 9 |
| [Ibrahim](https://www.semanticscholar.org/author/B.-Ibrahim/16787231) B. et al (2016)(94) | * | * | * | ** | * | ** | * | 9 |
| Awoleke J. et al (2019)(98) | * | * | * | * | * | ** | * | 8 |
| Ndeye S. et al (2021)(99) | * | * | * | ** | * | ** | * | 9 |
| Danyah S.et al (2021)(100) | * | * | * | ** | * | ** | * | 9 |
| Azeze G. et al (2020)(101) | * | * | * | * | ** | ** | * | 9 |
| [Inungu](https://www.semanticscholar.org/author/J.-Inungu/4039784) J. et al (2013)(102) | * | * | * | ** | * | ** | * | 9 |
| [Inungu](https://www.semanticscholar.org/author/J.-Inungu/4039784) J. et al (2013)(102) | * | * | * | * | * | ** | * | 8 |
| [Tesfahun T](https://www.semanticscholar.org/author/Tesfahun-Taddege-Geremew/150319285). et al(2021)(103) | * | * | * | ** | * | ** | * | 9 |
| [Akpan](https://www.semanticscholar.org/author/B.-Akpan/122181332) B. et al(2019)(104) | * | * | * | ** | * | ** | * | 9 |
| Waleligni A.et al(2020)(105) | * | * | * | ** | * | ** | * | 9 |
| Kandala N. et al (2019)(106) | * | * | * | * | * | ** | * | 8 |
| Théra T. et al (2015)(107) | * | * | * | ** | * | ** | * | 9 |
| Minsart F. et al (2014 )(108) | * | * | * | * | * | ** | * | 9 |
| Kandala B.et al (2015)(109) | * | * | * | ** | * | ** | * | 8 |
| Kandala B.et al (2015)(109) | * | * | * | ** | * | ** | * | 9 |
| Kaplan A. et al (2013)(110) |  |  |  |  |  |  |  |  |
| Sichone A. et al (2019)(111) | * | * | * | ** | * | ** | * | 9 |
| Bright O. et al (2013)(112) | * | * | * | ** | * | ** | * | 9 |
| Bright O. et al (2018)(112) | * | * | * | ** | * | ** | * | 9 |
| Duna A. et al (2017) (113) | * | * | * | * | ** | ** | * | 9 |
| Engelbert A. et al(2013)(114) | * | * | * | ** | * | ** | * | 9 |
| Edmund N. et al (2019)(115) | * | * | * | * | * | ** | * | 8 |
| Alphones K. et al (2019)(116) | * | * | * | ** | * | ** | * | 9 |
| [Mekuanint W](https://www.researchgate.net/profile/Mekuanint-Simeneh-Workie?utm_content=businessCard&utm_source=publicationDetail&rgutm_meta1=AC%3A21150225).et al (2020)(117) | * | * | * | ** | * | ** | * | 9 |
| Bjälkander O. et al (2013)(118) | * | * | * | ** | * | ** | * | 9 |
| Mohamed A. et al (2022) (119) | * | * | * | * | * | ** | * | 8 |
| [Talal A](https://www.researchgate.net/scientific-contributions/Talal-A-Abdel-Raheem-2139251812?_sg%5B0%5D=aLiwhE7QCfqPI3Gb8VyD_X3r9HyhRpgztPYJ15dz0W2QVSOAaiIhGRTo9NFbArHv0rtonFI.TRuKUsojW7luw7PRWmtImRXXKsaN6vACFmOhU-fkP9F6OwYrGl53XivqWBm4Vvsy379U5ikJdYbfDx-yl2JgLA.BmJ8Ryrw6MM_O9Kv1-nRvGoaCht2Bi7IzZRVN_HiUVRCrgdNmyA-BW9AiuyBbOGm46Yj0PFB8Kc-BajlrMVgCg&_sg%5B1%5D=fs4Rl7WcbdZNTLp-33m7AwGVuJd_o-3vIboNAOrLbWhrv2j0dk_9NfTJiJnPQCsABmXMNUE.uqxGrbsxlzxCxAc8ZG5gwvop2pzLuuBv8nYo61rc-Wgqx0Tjgsf9BOH9RMd2inQO7G1ag6j6RO1swCMTOlQuEw). et al (2018)(120) | * | * | * | ** | * | ** | * | 9 |
| Abdelmoneim K. et al (2017)(121) | * | * | * | * | * | ** | * | 9 |
| Onuchukwu V. et al (2017)(122) | * | * | * | ** | * | ** | * | 8 |
| Amal Y. et al (2019)(123) | * | * | * | ** | * | ** | * | 9 |
| Azeze G. et al (2020)(101) |  |  |  |  |  |  |  |  |
| Eman S. et al (2017)(124) | * | * | * | ** | * | ** | * | 9 |
| Gedion A. et al (2021)(101) | * | * | * | ** | * | ** | * | 9 |
| Gedion A. et al (2021)(101) | * | * | * | ** | * | ** | * | 9 |
| Aderibigbe S. et al (2018)(125) | * | * | * | * | ** | ** | * | 9 |
| Edmund O. et al (2019) (126) | * | * | * | ** | * | ** | * | 9 |

Table 2: Risk of bias assessment for cohort studies – Newcastle-Ottawa Scale (adaptation)

| **NOS scale for cohort studies** | **Selection** | | | | Comparability of Cohorts on the Basis of the Design or Analysis | **Outcome** | | | Total score |
| --- | --- | --- | --- | --- | --- | --- | --- | --- | --- |
|  | Representativeness of the sample | Selection of the Non-Exposed Cohort | Ascertainment of Exposure | Demonstration That Outcome of Interest Was Not Present at Start of Study |  | Assessment of the outcome | Was Follow-Up Long Enough for Outcomes to Occur | Adequacy of Follow Up of Cohorts |  |
| Yassin K. et al (2018)(97) | * | * | * | * | ** | * | * | * | 9 |
